# Discrete functional and mechanistic roles of chromodomain Y-like 2 (CDYL2) transcript variants in breast cancer growth and metastasis

**DOI:** 10.7150/thno.43744

**Published:** 2020-04-06

**Authors:** Li-Feng Yang, Fan Yang, Fang-Lin Zhang, Yi-Fan Xie, Zhi-Xiang Hu, Sheng-Lin Huang, Zhi-Min Shao, Da-Qiang Li

**Affiliations:** 1 Fudan University Shanghai Cancer Center and Shanghai Key Laboratory of Medical Epigenetics, International Co-laboratory of Medical Epigenetics and Metabolism, Ministry of Science and Technology, Institutes of Biomedical Sciences, Fudan University, Shanghai 200032, China; 2 Cancer Institute, Shanghai Medical College, Fudan University, Shanghai 200032, China; 3 Department of Oncology, Shanghai Medical College, Fudan University, Shanghai 200032, China; 4 Department of Breast Surgery, Shanghai Medical College, Fudan University, Shanghai 200032, China; 5 Shanghai Key Laboratory of Breast Cancer, Shanghai Medical College, Fudan University, Shanghai 200032, China

**Keywords:** Breast cancer, CDYL2, transcript variants, alternative splicing, transcriptional repression

## Abstract

**Rationale**: Chromodomain Y-like 2 (CDYL2) is a member of the CDY gene family involved in spermatogenesis, but its role in human cancer has not been reported. Analyses of publicly available databases demonstrate that CDYL2 is abundantly expressed in breast tumors. However, whether CDYL2 is involved in breast cancer progression remains unknown.

**Methods**: Quantitative real-time PCR and immunoblotting assays were used to determine the expression levels of CDYL2 transcript variants in breast cancer cell lines and primary breast tumors. The effect of CDYL2 transcript variants on the malignant phenotypes of breast cancer cells was examined through *in vitro* and *in vivo* assays. Immunofluorescent staining, RNA-seq, ATAC-seq, and ChIP-qPCR were used to investigate the underlying mechanisms behind the aforementioned observations.

**Results**: Here we show that CDYL2 generated four transcript variants, named CDYL2a-CDYL2d. CDYL2a and CDYL2b were the predominant variants expressed in breast cancer cell lines and breast tumors and exerted strikingly discrete functions in breast cancer growth and metastasis. CDYL2a was upregulated in the majority of the breast cancer cell lines and tumors, and promoted breast cancer cell proliferation, colony formation *in vitro*, and tumorigenesis in xenografts. In contrast, CDYL2b was mainly expressed in luminal- and HER2-positive types of breast cancer cell lines and tumors, and suppressed the migratory, invasive, and metastatic potential of breast cancer cells *in vitro* and *in vivo*. Mechanistically, CDYL2a partially localized to SC35-positive nuclear speckles and promoted alternative splicing of a subset of target genes, including FIP1L1, NKTR, and ADD3 by exon skipping. Elimination of full-length FIP1L1, NKTR, and ADD3 rescued the impaired cell proliferation through CDYL2a depletion. In contrast, CDYL2b localized to heterochromatin and transcriptionally repressed several metastasis-promoting genes, including HPSE, HLA-F, and SELL. Restoration of HPSE, HLA-F, or SELL expression in CDYL2b-overexpressing cells attenuated the ability of CDYL2b to suppress breast cancer cell migration and invasion.

**Conclusions**: Collectively, these findings establish an isoform-specific function of CDYL2 in breast cancer development and progression and highlight that pharmacological inhibition of the CDYL2a, but not the CDYL2b, isoform may be an effective strategy for breast cancer therapy.

## Introduction

Breast cancer is the leading cause of cancer death among females worldwide and is a highly heterogeneous disease 1. According to the expression status of the estrogen receptor α (ERα), progesterone receptor (PR), and human epidermal growth factor receptor 2 (HER2), breast cancer is clinically classified into three main subtypes, including luminal (ERα/PR-positive), HER2-positive, and triple-negative breast cancer (TNBC) 2. In general, luminal-type breast tumors have relatively good clinical outcomes with a lower chance of metastasis, whereas TNBC represents the most aggressive subtype of breast cancer with a higher incidence of early relapse, distant metastasis, and a worse prognosis 3, 4.

The phenotypic differences between distinct breast cancer subtypes are reflected in their diverse transcriptomic and proteomic profiles 5, 6. Evidence shows that the differential patterns of transcript variant expression are sufficient to differentiate breast cancer subtypes 5. Diverse transcript variants arise from alternative transcription initiation, splicing, and polyadenylation, and may give rise to distinct protein isoforms with diverse functions 7. The alternative transcription initiation, also known as alternative promoter usage, results in multiple pre-mRNAs being transcribed from different transcription start sites within a gene locus 8. This event may affect open reading frames in mRNAs and give rise to protein isoforms with different N-terminal sequences 9, 10. In the mammalian genome, over 50% of the genes are regulated through alternative transcription initiation (7). Alternative splicing is a two-step biological process, in which the introns are removed and then different exons of the same gene are selectively joined together to generate multiple transcripts 11. There are four main types of alternative splicing, including exon skipping, intron retention, mutually-exclusive exons, or alternative 5ʹ or 3ʹ splice site 12. It is estimated that approximately 95% of the human multiexon genes undergo alternative splicing 13. Alternative polyadenylation generates mRNAs with alternative 3' ends and therefore potentially regulates the stability, localization, and translation efficiency of target RNAs 14. Alternative transcription events are commonly deregulated in breast cancer, thus contributing to tumor initiation and progression, and providing potential therapeutic targets. For example, a switch from the CD44 variant isoform (CD44v) to the CD44 standard isoform (CD44s) is functionally essential for epithelial-mesenchymal transition and determines breast cancer stem cell state and metastasis 15-17. In addition, specific isoforms of IGF2BP2, NECTIN4, ITGB6, and KLHDC9 are associated with breast cancer cellular sensitivity to therapeutic agents AZD6244, lapatinib, erlotinib, and paclitaxel, respectively 18. Despite these important observations, the contribution of distinct transcript variants to breast cancer development and progression is not completely understood.

Chromodomain Y-like protein 2 (CDYL2) is a poorly characterized member of the chromodomain Y (CDY) protein family, which includes chromodomain Y (CDY) and chromodomain Y-like (CDYL) 19. This family of proteins is characterized by the presence of two conserved functional domains, an N-terminal chromodomain (CD) and a C-terminal enoyl-coenzyme A hydratase (ECH) catalytic domain 19, 20. The CD domain is present in multiple chromatin-binding proteins, such as the heterochromatin protein 1 (HP1), and is implicated in chromatin binding and the recognition of lysine-histone tails 21. The ECH domain is commonly found in coenzyme A (CoA)-dependent acylation enzymes, which are biochemically linked to fatty acid metabolism and are associated with cancer progression and drug resistance 22-24. Human CDY exhibits testis-specific expression, CDYL is ubiquitously expressed, whereas CDYL2 exhibits selective expression in tissues of testis, prostate, spleen, and leukocytes 19, 25. Evidence from human and mouse studies suggests that genes in the CDY family play an important role in spermatogenesis 26, 27. Recently, two RNA-sequencing (RNA-seq) based transcriptomic analyses demonstrated that CDYL2 is upregulated in CD133-positive colorectal cancer stem cells compared to CD133-negative counterparts 28, and that the exposure of rat serotonergic cells to valproic acid and lithium, two commonly used mood stabilizer drugs, results in downregulation of CDYL2 29. In addition, CDYL2 may affect susceptibility to inflammatory bowel disease in Koreans 30 and is potentially involved in osteoporosis pathogenesis 31. However, the functional and mechanistic role for CDYL2 in human cancer remains unexplored.

It is increasingly recognized that many of the spermatogenesis-related genes have crucial functions in tumorigenesis and tumor progression 32, 33. The analysis of publicly available databases demonstrates that CDYL2 not only is highly expressed in human testis tissues, but is also abundantly expressed in breast tumors. However, whether CDYL2 is involved in breast cancer development and progression remains unknown. In this study, we provide evidence for the first time that two CDYL2 transcript variants, named CDYL2a and CDYL2b, are predominantly expressed in human breast cancer cell lines and primary breast tumors and exert discrete functions in breast cancer growth and metastasis. These findings uncover an isoform-specific function of CDYL2 in breast cancer and highlight the importance of targeting key driver genes at the isoform level for breast cancer therapy.

## Methods

### Cell culture and reagents

Human mammary epithelial cell line (HMEC), human embryonic kidney cell line (HEK293T), and eleven breast cancer cell lines used in this study were obtained from the Type Culture Collection of the Chinese Academy of Sciences (Shanghai, China). All cell lines were authenticated by routine detection of morphology, cell vitality, DNA fingerprinting, and mycoplasma, and were kept in culture for less than six months after receipt. SK-BR-3 and MDA-MB-468 cells were cultured in McCoy's 5A (#L630) and Leibovitz's L-15 (#L620) media (BasalMedia, Shanghai, China), respectively. Other cell lines were cultured in DMEM medium (#L110, BasalMedia). All culture media were supplemented with 10% fetal bovine serum (#FSS500, ExCell Biol, Shanghai, China) and 1% penicillin-streptomycin (#S110B, BasalMedia).

### Human tissue specimens

Primary breast tumor samples and adjacent noncancerous breast tissues from patients diagnosed with invasive breast carcinoma were obtained from the Tissue Bank at Fudan University Shanghai Cancer Center under institutional guidelines. All experimental procedures were carried out according to The Code of Ethics of the World Medical Association (Declaration of Helsinki). Informed consent was obtained from all patients prior to surgery. Immunohistochemistry evaluation of ERα, PR, and HER2 expression status was conducted as part of the routine diagnostic procedure to determine molecular subtypes of breast cancer at the Department of Pathology of Fudan University Shanghai Cancer Center.

### DNA constructs, siRNAs, and transfection

CDYL2b cDNA was obtained from Vigene Bioscience (#CH869225, Rockville, MD, USA) and subcloned into the lentiviral vector pCDH-CMV-MCS-EF1-Puro (#CD510B-1, System Biosciences, Palo Alto, CA, USA) or pLVX-IRES-Neo (#632181, Biofeng, Shanghai, China) to generate pCDH-Flag-CDYL2b or pLVX-Flag-CDYL2b. We amplified the common sequence between CDYL2a and CDYL2b and synthesized the unique 5' sequence of CDYL2a (HuaGen Biotech, Shanghai, China). Then, we ligated those two sequences into pCDH-CMV-MCS-EF1-Puro or pLVX-IRES-Neo vector to produce Flag-CDYL2a using ClonExpress II One Step Cloning Kit (#C112-02, Vazyme Biotech, Nanjing, China). The short hairpin RNA (shRNA) sequences targeting human CDYL2 were obtained from BLOCK-iT RNAi Designer (http://rnaidesigner.thermofisher.com/ rnaiexpress/) and then cloned into pLKO.1-TRC vector (#10878, Addgene, Watertown, MA, USA). The cDNAs of HPSE (#CH806645), HLA-F (#CH864022) and SELL (#CH832492) were purchased from Vigene Bioscience. The detailed information of expression vectors and primers used in molecular cloning is provided in **Tables S1 and S2**. All small interfering RNAs (siRNAs) and negative control siRNA (siNC) were purchased from Ribobio (Beijing, China) and their target sequences are listed in **Table S3**.

To generate stable cell lines, plasmid constructs or shRNAs in lentiviral expression vectors were transfected into HEK293T together with packaging plasmid mix using Neofect DNA transfection reagent (#TF20120, TengyiBio, Shanghai, China). The supernatant containing viruses was harvested 48 h after transfection and used to infect cells in the presence of 8 μg/mL of polybrene (#H9268, Sigma-Aldrich, St. Louis, MO, USA). After 24 h of infection, cells were selected with 2 μg/mL of puromycin (#13884-500, Cayman Chemical, Ann Arbor, MI, USA). The siRNAs were transfected into cells using Lipofectamine 2000 (#11668019, Invitrogen, Carlsbad, CA, USA) at a final concentration of 50 nM.

### RNA extraction, RT-PCR, and qPCR

Total RNA was isolated from cultured cells and tissue samples using TRIzol reagent (#15596018, Invitrogen) and cDNA was synthesized using PrimeScript RT Master Mix (#RR036A, Takara, Dalian, China). PrimeSTAR Max DNA polymerase (#RR045A, Takara) was used for conventional RT-PCR assays. PCR products were separated by electrophoresis on a 2% agarose gel or subjected to sequencing. Quantitative real-time PCR (qPCR) was performed in triplicates using TB Green Premix Ex Taq (#RR420A, Takara) on an Eppendorf Realplex qPCR machine. All the primers were synthesized by HuaGene Biotech and their sequences are available in **Table S4**. The 2^-ΔΔCT^ method was used to determine the relative detection level of each mRNA in cell lines and clinical tissue samples 34.

### Chromatin immunoprecipitation-quantitative PCR (ChIP-qPCR)

SimpleChIP Plus Sonication Chromatin IP (#56383S, Cell Signaling Technology, Danvers, MA, USA) was used for ChIP assays according to the manufacturer's protocol. Approximately 1 × 10^7^ cells were cross-linked in 1% formaldehyde for 10 min at room temperature and quenched in glycine for 5 min. The solubilized chromatin was incubated with antibodies against Flag and IgG. The ChIP-qPCR primers are listed in **Table S4**.

### RNA-seq and ATAC-Seq

RNA-seq was performed as described previously 35. Briefly, RNA-seq library construction was performed using VAHTS mRNA-seq V2 Library Prep Kit for Illumina (#NR601-01, Vazyme), followed by sequencing using the Illumina sequencing platform. Sequenced reads were aligned to the hg38 genome assembly using HISAT2 software. Analyses for differential gene expression and alternative splicing were performed with DESeq2 and MISO as described previously 35. Assay for Transposase-Accessible Chromatin with high-throughput sequencing (ATAC-seq) was performed according to a published protocol 36. Briefly, about 1 × 10^5^ cells were washed with PBS and lysed with lysis buffer (10 mM Tris-Cl, pH 7.4, 10 mM NaCl, 3 mM MgCl_2_, 0.1% NP-40, 1X protease inhibitor). Then, the nuclei were resuspended in 25 μL TD buffer, 24 μL lysis buffer, and 1 μL TDE1 enzyme for 30 min at 55 °C. DNA was extracted and sequenced.

### Antibodies, immunoblotting, immunoprecipitation, and immunofluorescent staining

All antibodies used in this study are listed in **Table S5**. For immunoblotting analysis, cells were lysed in RIPA buffer. Equal amounts of proteins were separated by SDS-PAGE and transferred onto a PVDF membrane (#IPVH00010, Millipore, Billerica, MA, USA). The membranes were incubated with the appropriate primary antibodies and detected with the enhanced chemiluminescence detection kit (#36208ES80, Yeasen, Shanghai, China). For immunoprecipitation (IP) assays, cellular extracts were incubated with primary antibodies in a rotating incubator at 4°C overnight, followed by incubation with protein A/G magnetic beads (#B23202, Bimake, Houston, TX, USA) for another 2 h. The immunoprecipitate was washed three times and analyzed through immunoblotting. For immunofluorescent staining, cells were fixed with 4% paraformaldehyde, premeabilized in 0.1% Triton X-100, and blocked with 10% normal goat serum in PBST. Then, cells were incubated with the corresponding primary antibodies, washed three times using PBST, and incubated with second antibodies conjugated with Alexa Fluor 555 (#4409S or #4413S, Cell Signaling Technology) or Alexa Fluor 488 (#4408S or #4412S, Cell Signaling Technology). DNA was stained with fluoroshield mounting medium with DAPI (#ab104139, Abcam, Cambridge, MA, USA). Leica SP5 confocal laser scanning microscope (Leica Microsystems, Wetzlar, Germany) was used for imaging.

### Cell proliferation and colony formation assays

Cell proliferation assays were conducted using the Cell Counting Kit-8 (CCK-8) (#CK04, Dojindo Laboratories, Kumamoto, Japan). For colony formation assays, 1000 cells (8000 cells for BT20 cell line) were plated into 6-well plates in triplicates and cultured in normal conditions for 2 weeks. Colonies were fixed in methanol and stained with 0.5% crystal violet. Colonies with more than 50 cells were counted.

### Wound-healing, cell migration, and invasion assays

For wound-healing assays, cells were seeded in 6-well plates and a scratch was made using a 200 μL pipette tip. Cells were then washed with PBS and incubated in medium containing 0.1% FBS for the indicated times. For migration and invasion assays, 2 × 10^4^ cells in serum-free media were plated in the upper chambers coated with (invasion) or without (migration) growth factor-reduced Matrigel (#354480 and #353097, respectively, Corning, New York, NY, USA). After 24 h or 48 h, cells in the lower membranes of transwell chambers were fixed in methanol, stained with 0.5% crystal violet, and counted under a microscope.

### Xenograft tumors in nude mice

All animal experiments were performed in compliance with the guidelines of the Institutional Animal Care and Use Committee at Fudan University. For subcutaneous inoculation, 5×10^6^ MDA-MB-231 cells stably expressing pCDH, Flag-CDYL2a, or Flag-CDYL2b in 200 μL PBS were injected subcutaneously into the mammary fat pad of 6-week-old BALB/c female nude mice (State Key Laboratory of Oncogenes and Related Genes, Shanghai Cancer Institute, Shanghai, China). The tumor volume was measured twice a week and calculated with the formula (length×width^2^)/2. The mice were killed eight weeks later and tumors were removed and weighed individually. For experimental lung metastasis assays, 1.5 × 10^6^ MDA-MB-231 cells stably expressing pCDH, Flag-CDYL2a, or Flag-CDYL2b in 200 μL PBS were injected into the tail veins of 6-week-old BALB/c female nude mice. Six weeks after the injection, mice were sacrificed, the lungs were removed, and metastatic nodules were counted.

### Statistical analyses

All data are presented as the mean ± standard error from at least three independent experiments unless otherwise indicated. The unpaired two-tailed Student's *t* test was used to compare data between two groups. *P* values of less than 0.05 were considered statistically significant.

## Results

### CDYL2a and CDYL2b are the predominant isoforms expressed in breast cancer cell lines and tumors

In support of its potential role in spermatogenesis, analyses of the Human Protein Atlas (HPA) database 37 revealed that CDYL2 was primarily expressed in human testis, placenta, and prostate tissues, with extremely high expression levels in the testis (**Figure S1**). As many of spermatogenesis-related genes are reactivated in human cancer 32, 33, we further examined the expression patterns of CDYL2 in breast cancer. Analyses of multiple publicly available databases, including ONCOMINE 38, Expression Profiling Interactive Analysis (GEPIA) 39, and The Cancer Genome Atlas (TCGA) 40, revealed that the mRNA levels of CDYL2 were significantly higher in breast tumor tissues than those in normal breast tissues (**Figure S2-4**). Analyses of The Clinical Proteomic Tumor Analysis Consortium (CPTAC) database 41 also revealed that the protein levels of CDYL2 were upregulated in breast tumors relative to normal breast tissues (**Figure S5**). These bioinformatic analyses data prompted us to further investigate whether CDYL2 is involved in breast cancer development and progression.

Human CDYL2 pre-mRNA contains ten exons and generates four transcript variants, named CDYL2a (GenBank Accession No. XM_011522866.1), CDYL2b (NM_152342.4), CDYL2c (XM_011522867.2), and CDYL2d (XM_024450151.1), which share the last six exons (exons 5-10) with only differences in their first exons (**Figure S6A**). Those four transcripts encode four different protein isoforms, which consist of 540, 506, 503, and 447 amino acid residues with a predicted molecular weight of 59.4, 55.7, 55.3, and 49.2 kDa, respectively (**Figure S6B**). CDYL2a, CDYL2b, and CDYL2c proteins contain intact CD domain and ECH domain but differ in their N-terminal sequences. The CDYL2d isoform remains the C-terminal ECH domain but lacks the N-terminal CD domain (**Figure S6B**). Close inspection of the genomic and protein sequence revealed that the CDYL2b transcript variant corresponds to the CDYL2 cDNA and that the CDYL2b protein isoform corresponds to the CDYL2 protein documented in UniProtKB database (Q8N8U2).

**Figure 1:**
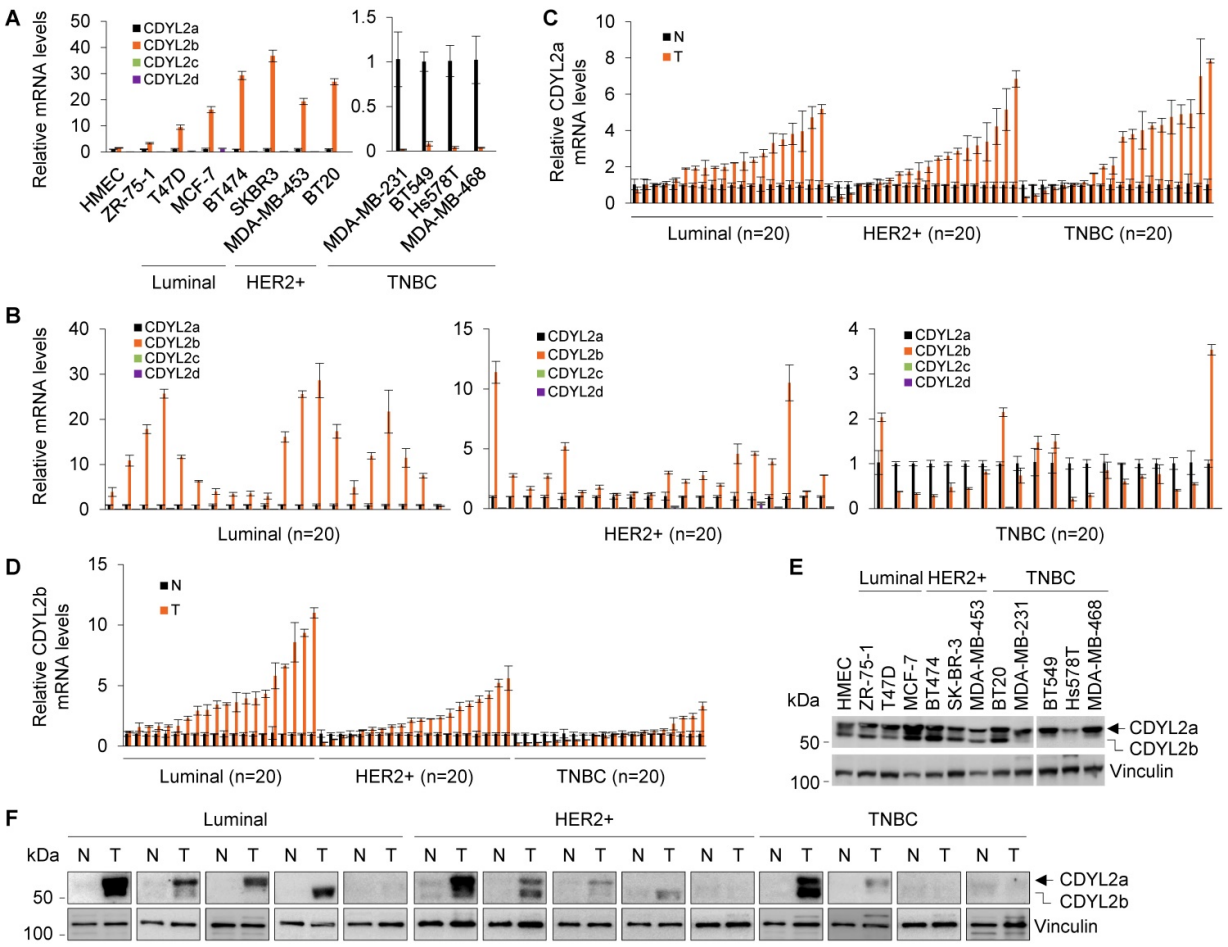
** CDYL2a and CDYL2b are the predominant isoforms expressed in human breast cancer cell lines and breast tumors.** (A-B) qPCR analysis of the relative mRNA expression levels of four CDYL2 transcription variants in the indicated cell lines (A) and 60 primary breast tumor samples (20 samples for luminal, HER2-positive, and TNBC subtype, respectively). The relative expression levels of CDYL2a, CDYL2b, CDYL2c, and CDYL2d mRNA were first normalized to the GAPDH levels. Then, CDYL2a was used as a control in each breast cancer cell line (A) and breast tumor sample (B) as it was detectable in all samples. (C-D) qPCR analysis of the relative mRNA expression levels of CDYL2a (C) and CDYL2b (D) in 60 pairs of primary breast tumor tissues and matched normal breast tissues (20 samples for luminal, HER2-positive, and TNBC subtype, respectively). The expression levels of CDYL2a (C) or CDYL2b (D) in normal tissues were used as a control after normalization to the GAPDH levels. (E) Immunoblotting analysis of CDYL2 protein expression in the indicated cell lines with an anti-CDYL2 antibody. Vinculin was used as a loading control. (F) Immunoblotting analysis of CDYL2 protein expression in 14 pairs of primary breast tumor tissues and matched normal breast tissues with an anti-CDYL2 antibody. Vinculin was used as a loading control.

To examine the expression pattern of those four transcripts in human breast cancer cell lines and tumor tissues, we first performed qPCR analyses using isoform-specific primers. Results showed that the human mammary epithelial cell line HMEC and eleven breast cancer cell lines primarily expressed CDYL2a and CDYL2b isoforms (**Figure 1A**). In contrast, CDYL2c and CDYL2d isoforms were barely detectable in these cell lines, suggesting that those two isoforms, if they are present, do not play a prominent role in breast cancer pathogenesis. In addition, we noticed that the expression levels of CDYL2b were significantly lower in the majority of TNBC cell lines (with the exception of BT20 cell line) than those in luminal and HER2-positive breast cancer cell lines 42 (**Figure 1A**). To further validate these results, conventional RT-PCR analysis was performed using RNA extracted from MCF-7 cells and the resultant PCR products were subjected to agarose gel electrophoresis and sequencing. Results showed that only CDYL2a and CDYL2b cDNAs were amplified in MCF-7 cells (**Figure S7A**). Sequencing of the PCR products further demonstrated that the sequences of CDYL2a and CDYL2b were identical to the ones documented in the NCBI database (**Figure S7B-C**).

Next, we examined the expression status of the four CDYL2 transcripts in sixty pairs of primary breast tumor tissues and adjacent noncancerous breast tissues (twenty pairs for luminal, HER2-positive, and TNBC subtypes, respectively) and found that primary breast tumors mainly expressed CDYL2a and CDYL2b (**Figure 1B**). Moreover, CDYL2a was upregulated in the majority of the breast tumors relative to matched normal breast tissues (**Figure 1C**). In contrast, CDYL2b was mainly upregulated in luminal and HER2-positive breast tumors relative to TNBC tumors (**Figure 1D**), which is consistent with the expression pattern of CDYL2 in the TCGA database (**Figure S4C**).

To analyze the protein expression levels of different CDYL2 isoforms in human breast cancer cell lines and breast tumors, we conducted immunoblotting analyses using a commercial CDYL2 antibody (#ab183854, Abcam). This antibody was generated using a recombinant fragment within the human CDYL2 aa 1-351 and the exact sequence is proprietary. As the CDYL2a protein contains the same N-terminal 351 aa as CDYL2b does (**Figure S6B**), this CDYL2 antibody is able to recognize both CDYL2a and CDYL2b proteins. As shown in **Figure 1E**, two main bands were observed in HMEC and eleven breast cancer cell lines. According to the predicted molecular weight, the upper and lower bands corresponded to CDYL2a (about 59.4 kDa) and CDYL2b (about 55.7 kDa), respectively. This conclusion was further confirmed by subsequent experiments with overexpression of CDYL2a and CDYL2b. In addition, we noticed that CDYL2a protein levels were upregulated in most breast cancer cell lines. In contrast, CDYL2b was upregulated in luminal-type and HER2-positive breast cancer cell lines, but was downregulated in most TNBC cell lines compared to the HMEC cell line (**Figure 1E**). In fourteen pairs of clinical samples, CDYL2a and CDYL2b were upregulated in 57.1% (8/14) and 35.7 % (5/14) of the primary breast tumors relative to matched normal breast tissues, respectively (**Figure 1F**). Collectively, these results suggest that breast cancer cell lines and breast tumor tissues primarily express CDYL2a and CDYL2b.

### CDYL2a plays a growth-promoting role, whereas CDYL2b exerts an antimetastatic role in breast cancer progression

**Figure 2:**
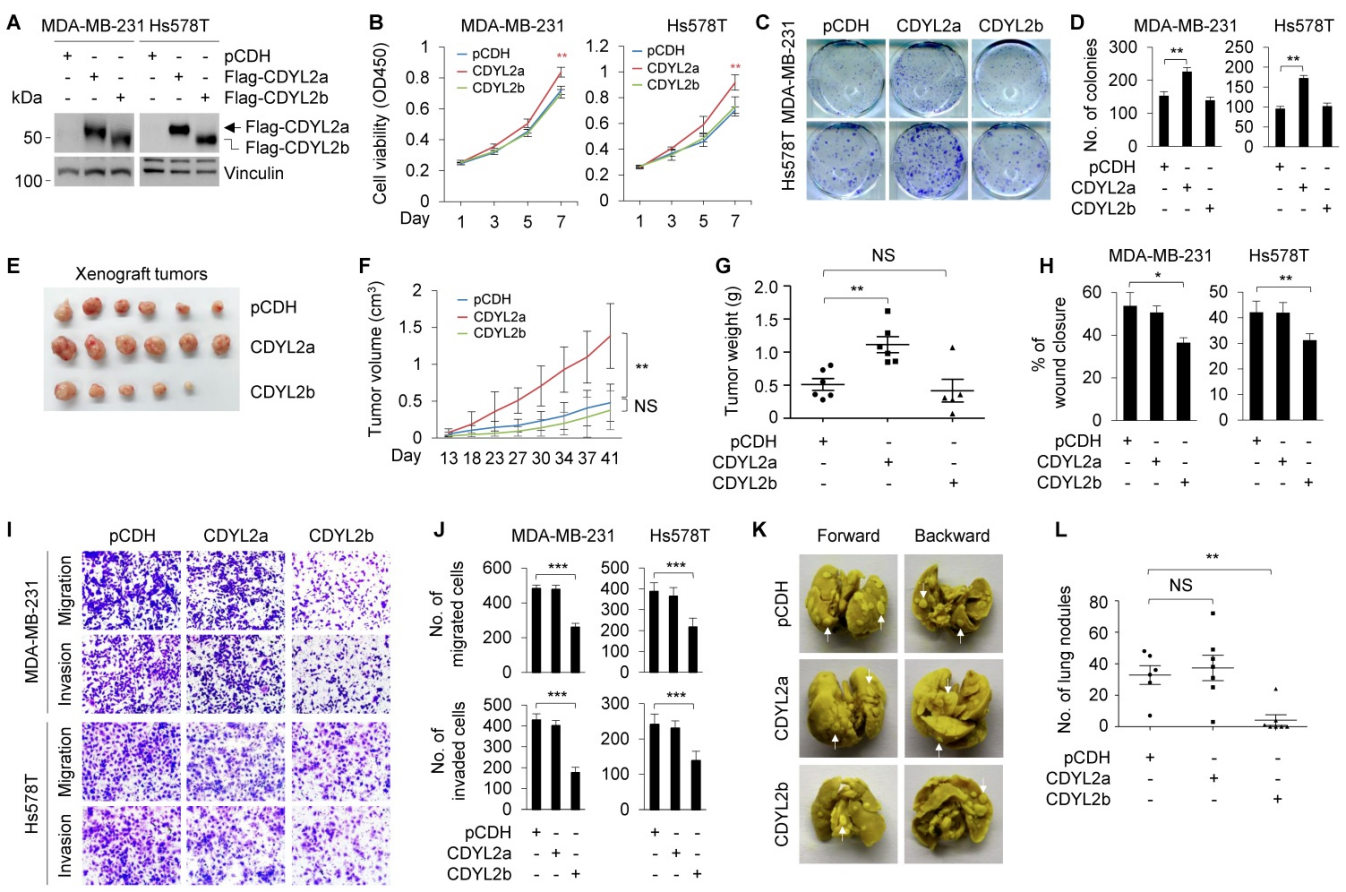
** CDYL2a promotes breast cancer cell proliferation and tumor growth while CDYL2b suppresses breast cancer cell migratory, invasive, and metastatic potential.** (A) Establishment of stable MDA-MB-231 and Hs578T cells expressing pCDH, Flag-CDYL2a, and Flag-CDYL2b by lentiviral infection. The expression status of Flag-CDYL2a and Flag-CDYL2b in these cell lines was validated by immunoblotting analysis with an anti-Flag antibody. Vinculin was used as a loading control. (B-D) MDA-MB-231 and Hs578T cells stably expressing pCDH, Flag-CDYL2a, or Flag-CDYL2b were subjected to cell proliferation assays using CCK-8 (B) and colony formation assays (C-D). Representative images of the colonies (C) and quantitative results (D) are shown. (E-G) MDA-MB-231 cells stably expressing pCDH, Flag-CDYL2a, or Flag-CDYL2b were injected into mammary fat pads of 6-week-old female BALB/c nude mice (n = 6). After 8 weeks of injections, xenograft tumors were harvested. Photographs of harvested tumors (E), tumor growth curves (F), and tumor weights (G) are shown. (H-J) MDA-MB-231 and Hs578T cells stably expressing pCDH, Flag-CDYL2a, or Flag-CDYL2b were subjected to wound-healing assays (H), Boyden's chamber migration assays and Matrigel-coated invasion assays (I and J). Representative images of the wound-healing assays are included in Supplementary Figure S8A and the corresponding quantitative results are shown in H. Representative images of Transwell migration and invasion assays are shown in I and the corresponding quantitative results are shown in J. (K-L) MDA-MB-231 cells stably expressing pCDH, Flag-CDYL2a, or Flag-CDYL2b were injected into 6-week-old female BALB/c nude mice (n = 6) through the tail vein. After 6 weeks of injections, the lungs were harvested. Representative images of lung metastasis (K) and quantitative results of lung nodules (L) are shown. NS, are not significant differences; *, **, and *** are significant differences at *p*<0.05, *p*< 0.01, and *p*<0.001 levels, respectively.

To delineate the biological functions of CDYL2a and CDYL2b in breast cancer development and progression, we stably expressed Flag-CDYL2a and Flag-CDYL2b in MDA-MB-231 and Hs578T cells, which express relatively low levels of endogenous CDYL2a and CDYL2b (**Figure 1E**), through lentiviral infection. The expression status of CDYL2a and CDYL2b in those two cell lines was determined by immunoblotting with an anti-Flag antibody (**Figure 2A**). CCK-8 (**Figure 2B**) and colony formation (**Figure 2C-D**) assays demonstrated that the ectopic expression of CDYL2a enhanced the proliferation and colony formation capability of MDA-MB-231 and Hs578T cells compared to empty vector controls. In contrast, overexpression of CDYL2b did not have this effect (**Figure 2B-D**). To investigate whether CDYL2a and CDYL2b might regulate tumorigenic capacity of breast cancer cells *in vivo*, MDA-MB-231 cells stably expressing the empty vector pCDH, Flag-CDYL2a, or Flag-CDYL2b were subcutaneously injected into mammary fat pads of 6-week-old female BALB/c nude mice. In support of *in vitro* findings, tumors from CDYL2a overexpressing MDA-MB-231 cells grew much faster at the implantation sites than in their control cells (**Figure 2E**); this faster growth was accompanied with an increase in the tumor volume (**Figure 2F**) and weight (**Figure 2G**). This phenomenon was not observed in tumors from CDYL2b overexpressing cells (**Figure 2E-G**). Collectively, these results suggest that CDYL2a promotes breast cancer cell proliferation and colony formation *in vitro* and xenograft tumor growth *in vivo*.

As one of the key features of breast cancer cells is their invasive and metastatic behavior 43, we further examined whether CDYL2a and CDYL2b might modulate the invasive and metastatic phenotype of breast cancer cells. Wound-healing assays showed that CDYL2b overexpressing MDA-MB-231 and Hs578T cells had a lower closure of the wound area compared to that of the control cells (**Figures 2H and S8A**). In contrast, the ectopic expression of CDYL2a did not significantly affect cell migration (**Figures 2H and S8A**). These results were further confirmed with Boyden's chamber migration assays (**Figure 2I-J**). Moreover, MDA-MB-231 and Hs578T cells stably expressing CDYL2b showed a reduced invasion in Matrigel-coated invasion chambers (**Figure 2I-J**). These results indicate that CDYL2b, but not CDYL2a, suppresses breast cell migration and invasion *in vitro*. To examine whether CDYL2b affects the metastatic potential of breast cancer cells *in vivo*, MDA-MB-231 cells stably expressing the empty vector pCDH, Flag-CDYL2a, or Flag-CDYL2b were injected into nude mice through tail veins. As shown in **Figure 2K-L**, CDYL2b-overexpressing cells significantly decreased the number of metastatic tumors in the lungs of mice as compared with that of empty vector-expressing cells. Collectively, these results establish that CDYL2b suppresses breast cancer cell migration and invasion *in vitro* and the formation of lung metastases in experimental lung metastasis mouse models.

As the CDYL2b transcript variant corresponds to the CDYL2 cDNA, we analyzed the relation between CDYL2 expression levels and the prognosis in patients with breast cancer *via* the Kaplan-Meier plotter database 44. Results showed that high expression of CDYL2 was associated with better relapse-free survival (RFS) of all patients with breast cancer (**Figure S8B**). Subgroup analyses based on molecular subtypes of breast tumors showed that high CDYL2b expression was significantly associated with better RFS in patients with luminal A, luminal B, and basal-like tumors but not in HER2-positive ones (**Figure S8C**). These results highlight a discrete function of CDYL2b in different subtypes of breast cancers. It is noteworthy mentioning that currently no CDYL2a expression information is available in the Kaplan-Meier plotter database, thus we failed to analyze the association between CDYL2a expression levels and the prognosis of patients with breast cancer.

**Figure 3:**
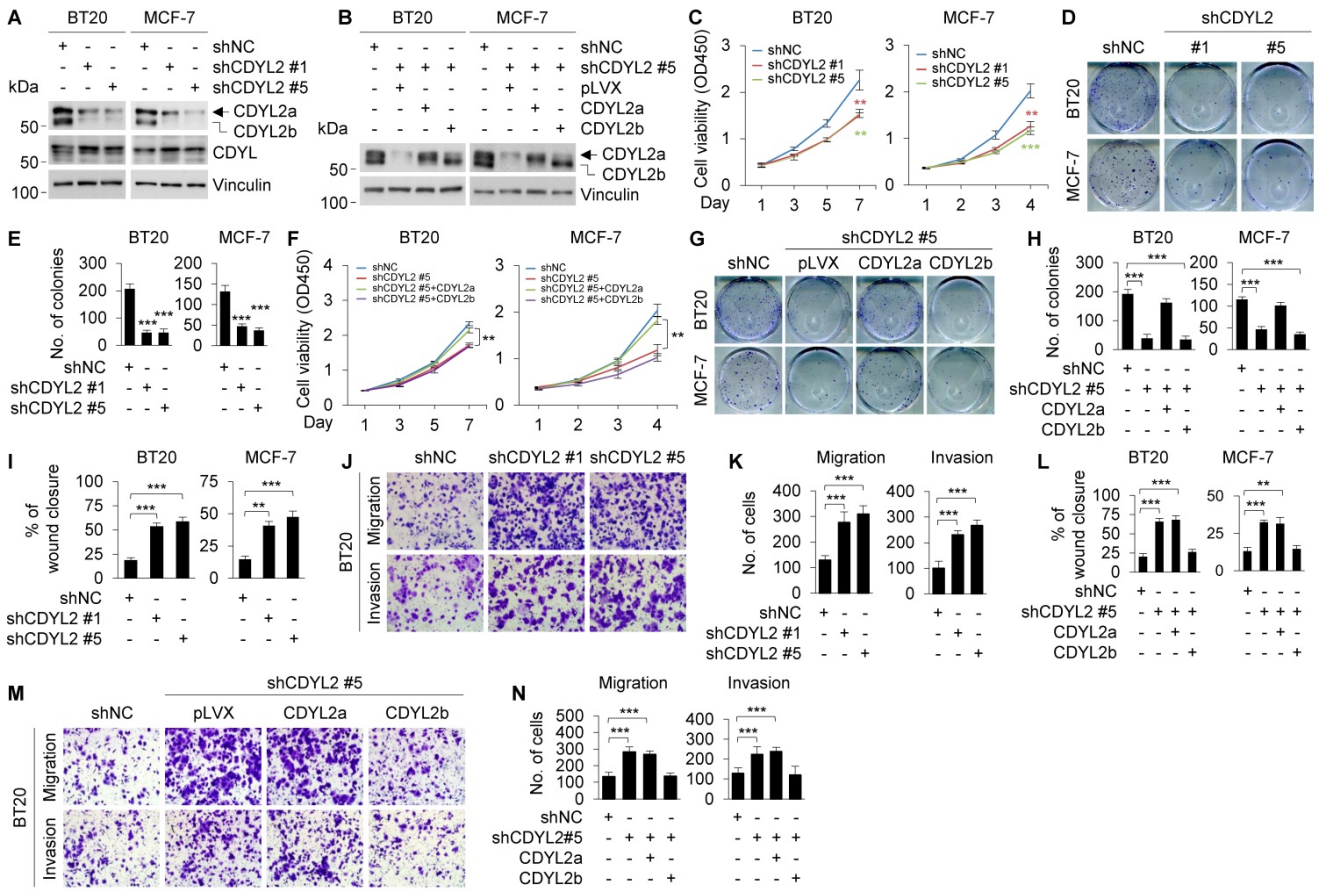
** Re-expression of CDYL2a restores the impaired cell proliferation of CDYL2-depleted cells, while re-expression of CDYL2b reverses the enhanced cell migratory and invasive potential of CDYL2-depleted cells.** (A) Immunoblotting analysis of CDYL2 protein expression in BT20 and MCF-7 cells stably expressing shNC, shCDYL2 #1, or shCDYL2 #5 with an anti-CDYL2 antibody. Vinculin was used as a loading control. (B) CDYL2-depleted BT20 and MCF-7 cells were reconstituted with Flag-CDYL2a or Flag-CDYL2b by lentiviral infection. The expression status of CDYL2a and CDYL2b in these cell lines was validated with immunoblotting analysis using an anti-CDYL2 antibody. Vinculin was used as a loading control. (C-E) BT20 and MCF-7 cells stably expressing shNC, shCDYL2 #1, or shCDYL2 #5 were subjected to cell proliferation assays using CCK-8 (C) and colony growth assays (D-E). Representative images of the colonies (D) and quantitative results (E) are shown. (F-H) CDYL2-depleted BT20 and MCF-7 cells were reconstituted with Flag-CDYL2a and Flag-CDYL2b by lentiviral infection and then subjected to cell proliferation assays using CCK-8 (F) and colony growth assays (G-H). Representative images of the colonies (G) and quantitative results (H) are shown. (I-K) BT20 and MCF-7 cells stably expressing shNC, shCDYL2#1, or shCDYL2#5 were subjected to wound-healing assays (I), Boyden's chamber migration assays and Matrigel-coated invasion assays (J-K). Representative images of wound-healing assays are shown in Supplementary Figure S9B and the corresponding quantitative results are shown in I. Representative images of Transwell migration and invasion assays are shown in J and the corresponding quantitative results are shown in K. (L-N) CDYL2-depleted BT20 and MCF-7 cells were reconstituted with Flag-CDYL2a and Flag-CDYL2b by lentiviral infection and then subjected to wound-healing assays (L), Boyden's chamber migration assays, and Matrigel-coated invasion assays (M-N). Representative images of wound-healing assays are shown in Supplementary Figure S9C and the corresponding quantitative results are shown in L. Representative images of Transwell migration and invasion assays are shown in M and corresponding quantitative results are shown in N. ** and *** are significant differences at *p*<0.01 and *p*<0.001, respectively.

To further confirm isoform-specific functions of CDYL2, we knocked down CDYL2 in BT20 and MCF-7 cell lines, both expressing CDYL2a and CDYL2b isoforms, by lentiviral infection with expression vectors encoding shRNA targeting CDYL2 (shCDYL2) and negative control shRNA (shNC). Worthy of note, we were not able to design specific shRNA sequences targeting only CDYL2a or CDYL2b due to a tiny discrepancy in the unique sequence of CDYL2a or CDYL2b mRNA. Immunoblotting analysis showed that shCDYL2 #1 and shCDYL2 #5 efficiently knocked down CDYL2a and CDYL2b in both cell lines, and the knockdown efficacy of shCDYL2 #5 was better than that of shCDYL2 #1 (**Figure 3A**). As a control, both shCDYL2 #1 and shCDYL2 #5 did not significantly affect the expression levels of CDYL, a closely related member of the CDY family 19 (**Figure 3A**). The knockdown effects mediated by shCDYL2 #1 and shCDYL2 #5 were also validated by qPCR analysis (**Figure S9A**). Moreover, we reconstituted CDYL2a and CDYL2b into CDYL2-depleted BT20 and MCF-7 cells by lentiviral infection, and selected the stable clones in which the levels of CDYL2a and CDYL2b overexpression were comparable to those of endogenous CDYL2a and CDYL2b in both cell lines for subsequent functional studies (**Figure 3B**). CCK-8 and colony formation assays revealed that the knockdown of CDYL2 suppressed cell proliferation and colony formation capacity of BT20 and MCF-7 cells (**Figure 3C-E**), and the effects were restored by re-expression of CDYL2a but not CDYL2b (**Figure 3F-H**). Cells expressing shCDYL2 exhibited higher migratory and invasive ability than shNC expressing control cells (**Figures 3I-K and S9B**). Moreover, shCDYL2-mediated increase in cell migration and invasion was compromised by the reconstitution of CDYL2b, but not CDYL2a, into cells expressing shCDYL2 (**Figures 3L-N and S9C**).

To further validate the above results, we repeated those assays in BT549 and HCC1806 cell lines, which predominately express CDYL2a. Results showed that the knockdown of CDYL2a (**Figure S10A-B**) resulted in a decrease in cell proliferation (**Figure S10C**) and colony formation capacity of BT549 and HCC1806 cells (**Figure S10D**), but did not significantly affect their migratory and invasive potential (**Figure S10E-F**). Collectively, these results demonstrated that CDYL2a promotes breast cancer cell proliferation, whereas CDYL2b suppresses breast cancer cell migratory and invasive behaviors.

### CDYL2a partially localizes to nuclear speckles while CDYL2b is localized in the heterochromatin

**Figure 4:**
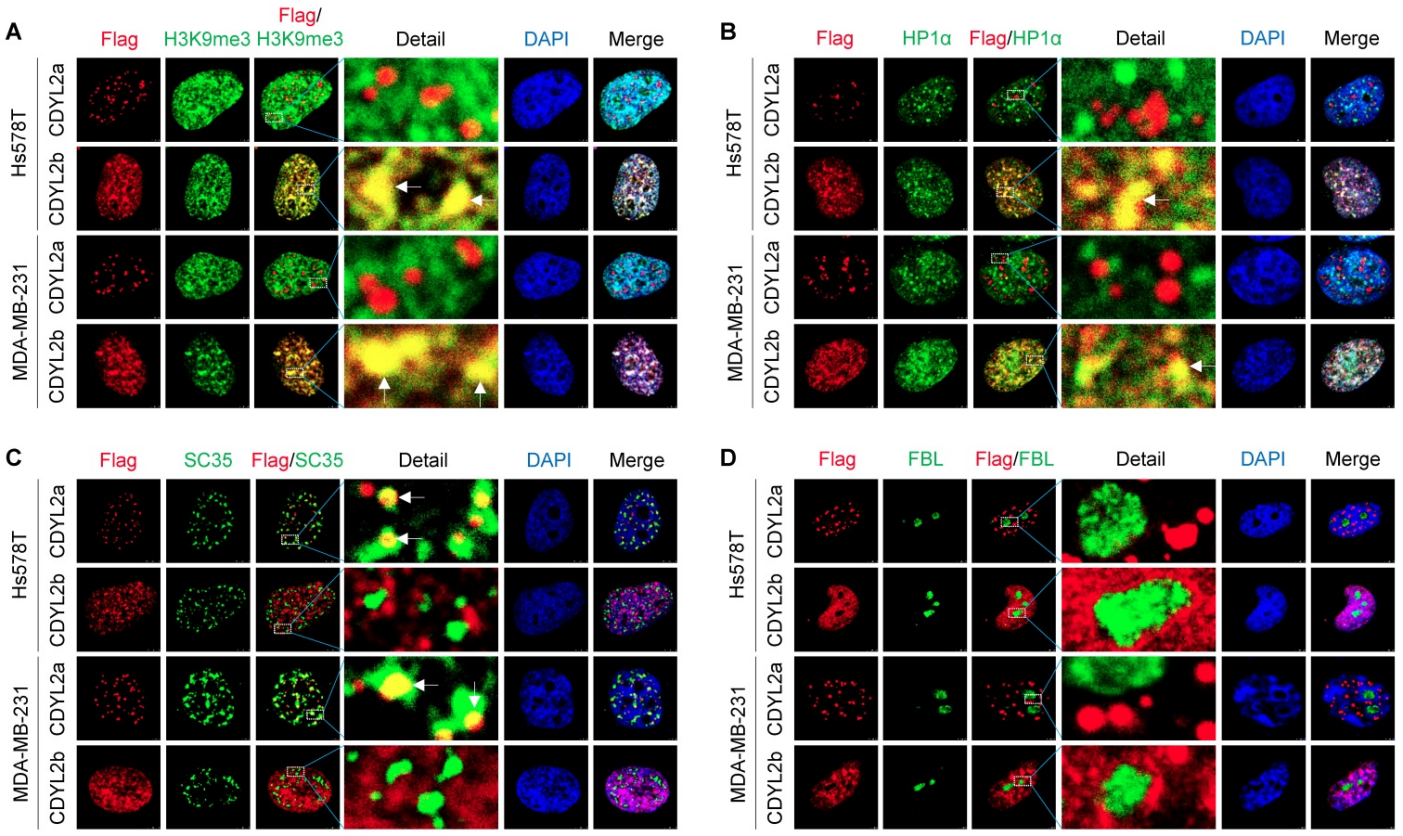
** Subcellular localization of CDYL2a and CDYL2b.** (A-D) MDA-MB-231 and Hs578T cells were transfected with plasmid DNAs encoding empty vector, pCDH, Flag-CDYL2a, or Flag-CDYL2b. After 48 h of transfection, cells were subjected to immunofluorescence staining with an antibody against Flag (red), H3K9me3 (A), heterochromatin protein 1α (HP1α) (B), SC35 (C), or fibrillarin (FBL) (D). DNA was counterstained with DAPI (blue).

Protein subcellular localization is intimately linked to protein functions in health and disease. To examine the subcellular localization of CDYL2a and CDYL2b, we transfected Flag-CDYL2a or Flag-CDYL2b into MDA-MB-231 and Hs578T cells and then conducted immunofluorescent staining with the appropriate antibodies. Results showed that both CDYL2a and CDYL2b were present in the nucleus but localized to distinct nuclear subcompartments. In this context, CDYL2b was co-localized with the histone H3 trimethylation at lysine 9 (H3K9me3) and the heterochromatin protein 1α (HP1α), two hallmarks of the heterochromatin (**Figure 4A-B**). This result is consistent with a recent report showing that CDYL2 co-localizes with H3K9me3 20. Immunoprecipitation (IP) assays further demonstrated that CDYL2b, but not CDYL2a, interacted with H3K9me3 (**Figure S11A**). In contrast, CDYL2a was distributed in a speckle-like pattern in the nucleus and did not co-localize with HP1α, H3K9me3, and DAPI (**Figure 4A-B**). In addition to chromatin, the nucleus contains several types of nuclear subcompartments (or nuclear bodies), such as nucleolus, nuclear speckles, promyelocytic leukemia (PML) bodies, paraspeckles, and Cajal bodies 45. Markers for those nuclear compartments have been well established and demonstrated to be applicable for co-localization studies in living cells 46. Further studies revealed that CDYL2a was partially co-localized with the spliceosomal protein SC35 (also known as serine-and-arginine-rich splicing factor 2, SRSF2), an established nuclear speckle marker 46 (**Figure 4C**). We also confirmed the interaction between CDYL2a and SC35 with IP assays (**Figure S11B**). In addition, CDYL2a did not localize to other nuclear bodies, as it lacked co-localization with the nucleolar proteins fibrillarin (FBL) and nucleophosmin (NPM) (**Figures 4D and S11C**), PML body marker PML protein (**Figure S11D**), paraspeckle marker non-POU domain-containing octamer-binding protein (NONO) (**Figure S11E**), and Cajal body formatting protein Coilin (**Figure S11F**). Collectively, these results demonstrated that CDYL2a and CDYL2b localize to distinct subnuclear compartments, which may be relevant to their nuclear functions.

### CDYL2a promotes breast cancer cell proliferation through regulating alternative splicing of FIP1L1, NKTR, and ADD3 genes

**Figure 5:**
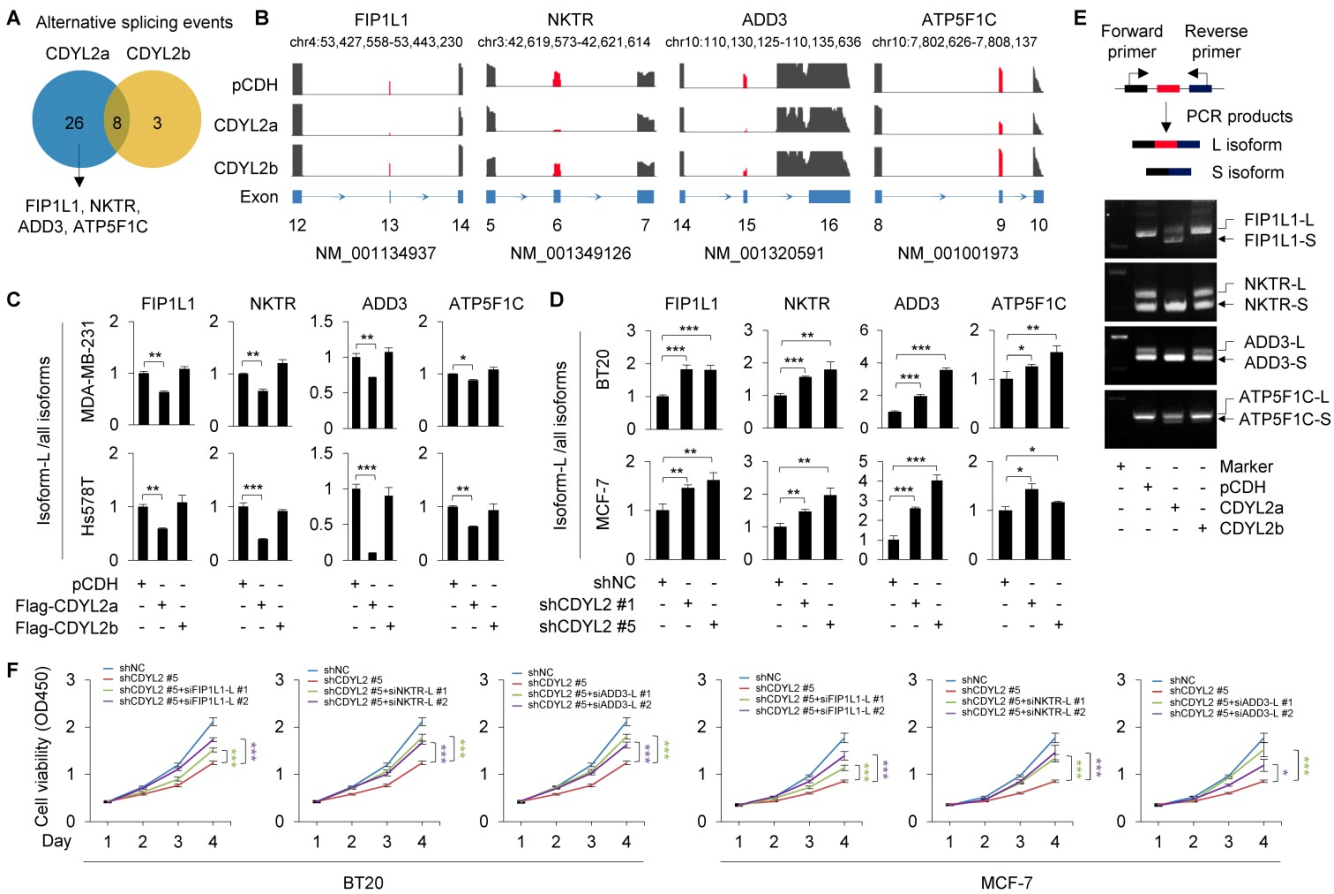
** CDYL2a promotes breast cancer cell proliferation through regulating alternative splicing of FIP1L1, NKTR, and ADD3 genes.** (A) Hs578T cells stably expressing pCDH, Flag-CDYL2a, or Flag-CDYL2b were subjected to RNA-seq analysis and alternative splicing events with FDR < 0.05 and absolute ΔPSI value > 0.1 are shown. (B) Exon skipping events within FIP1L1, NKTR, ADD3, and ATP5F1C genes are shown. (C) qPCR analysis was conducted with isoform-specific primers using MDA-MB-231 and Hs578T cells stably expressing pCDH, Flag-CDYL2a, or Flag-CDYL2b. The ratios of the L isoforms to all isoforms of FIP1L1, NKTR, ADD3, and ATP5F1C genes are shown. (D) qPCR analysis was performed with isoform-specific primers using BT20 and MCF-7 cells stably expressing shNC, shCDYL2#1, or shCDYL2#5. The ratios of the L isoforms to all isoforms of FIP1L1, NKTR, ADD3, and ATP5F1C genes are shown. (E) RT-PCR validation of individual splicing events regulated by CDYL2a. The cDNA from Hs578T cells stably expressing pCDH, Flag-CDYL2a, or Flag-CDYL2b were subjected to PCR amplification using primers flanking the alternative exon. (F) BT20 and MCF-7 cells stably expressing shNC and shCDYL2#5 were transfected with the indicated siRNAs targeting the L isoforms of FIP1L1, NKTR, or ADD3 gene. After 48 h of transfection, cells were subjected to cell proliferation assays using CCK-8 kit. The knockdown effect of transfected siRNAs targeting the L isoforms of FIP1L1, NKTR, ADD3 genes was validated by qPCR analysis and the corresponding results are shown in Figure S14. *, **, and *** are significant differences at *p*<0.05, *p*<0.01, and *p*<0.001 levels, respectively.

Nuclear speckle, also known as splicing speckle, is a subnuclear compartment enriched in small ribonucleoprotein particles and various splicing factors and is involved in splice site selection in both constitutive and alternative splicing 45. As CDYL2a is localized in SC35-positive nuclear speckles, we then examined whether CDYL2a affects alternative splicing events of its target genes. To do this, Hs578T cells stably expressing pCDH, Flag-CDYL2a, and Flag-CDYL2b were subjected to RNA-seq to define potential alternative splicing events. Using a cutoff of false discovery rate (FDR) < 5% and absolute ΔPSI (percentage of spliced in) value > 0.1, we identified thirty-four and eleven alternative splicing events in CDYL2a- and CDYL2b-overexpressing cells, respectively (**Figure 5A**). The main type of these identified splicing events was exon skipping. According to absolute ΔPSI value, we selected four genes that were detected in CDYL2a overexpressing cells with remarkable exon skipping for further validation, including the factor interacting with PAPOLA and CPSF1 (FIP1L1), the natural killer cell triggering receptor (NKTR), adducin 3 (ADD3), and the ATP synthase F1 subunit gamma (ATP5F1C) (**Figure 5B**). For convenience of description, we referred to the longer isoform of those four genes as L and to the shorter isoform as S. The skipping of FIP1L1 (exon 13), NKTR (exon 6), ADD3 (exon 15), and ATP5F1C (exon 9) was further demonstrated by qPCR with exon-specific primers, as the ratio of L isoforms to all isoforms (L plus S isoforms) of those genes was consistently downregulated in CDYL2a-overexpressing MDA-MB-231 and Hs578T cells (**Figure 5C**). In contrast, overexpression of CDYL2a did not affect the total isoform levels of FIP1L1, NKTR, ADD3, and ATP5F1C (**Figure S12**). In support of the above results, knockdown of CDYL2 with two different shRNAs resulted in upregulation of L isoforms of FIP1L1, NKTR, ADD3, and ATP5F1C in BT20 and MCF-7 cells (**Figure 5D**). Furthermore, the isoform switch of FIP1L1, NKTR, ADD, and ATP5F1C from L isoforms to S isoforms was further demonstrated by conventional RT-PCR in Hs578T cells (**Figure 5E**). We then detected those four splicing events in primary breast tumors, which highly expressed CDYL2a (**Figure 1D**). Results showed that the exon skipping events of FIP1L1, NKTR, and ADD3, but not ATP5F1C, occurred in most tumor tissues highly expressing CDYL2a compared to matched normal breast tissues (**Figure S13A**). Following these observations, we analyzed the exon skipping events of FIP1L1, NKTR, and ADD3 in tumor samples from the above xenograft tumor models (**Figure 2E**). Overexpression of CDYL2a resulted in a decrease in the L isoform levels of FIP1L1, NKTR, and ADD3 genes (**Figure S13B**).

FIP1L1 is a component of the cleavage and polyadenylation specificity factor (CPSF) and is associated with two leukemogenic fusion genes, the FIP1L1-retinoic acid receptor alpha (RARA) and the FIP1L1-platelet-derived growth factor receptor alpha (PDGFRA), thus contributing to the pathogenesis of distinct types of leukemia 47, 48. In addition, genetic alterations of FIP1L1 are associated with survival of patients with gliomas 49. NKTR is required for natural killer-like activity in human T cells 50 and is supposed to be involved in tumor growth inhibition in a SP2/0 myeloma model 51. ADD3 is a subunit of adducins 52 and its alternative splicing events have been reported in HER2-positive breast cancer 53 and in non-small cell lung cancer 54. A recent study revealed that miR-145 may suppress the activity of ADD3 and inhibit the proliferation of glioblastoma cells 55. To test whether CDYL2a enhances breast cancer proliferation through regulating the levels of the L isoforms of those three genes, we knocked down the L isoforms of the genes by siRNAs (**Figure S14A-C**). CCK-8 cell proliferation assays showed that knockdown of the L isoforms of FIP1L1, NKTR, and ADD3 might compromise shCDYL2-mediated inhibitory effects on cell proliferation of both BT20 and MCF-7 cells (**Figure 5F**). Collectively, these results suggest that CDYL2a promotes breast cancer cell proliferation through, at least partly, regulating the alternative splicing of FIP1L1, NKTR, and ADD3 genes.

### CDYL2b suppresses breast cancer cell migration and invasion through transcriptional inhibition of several metastasis-promoting genes

**Figure 6:**
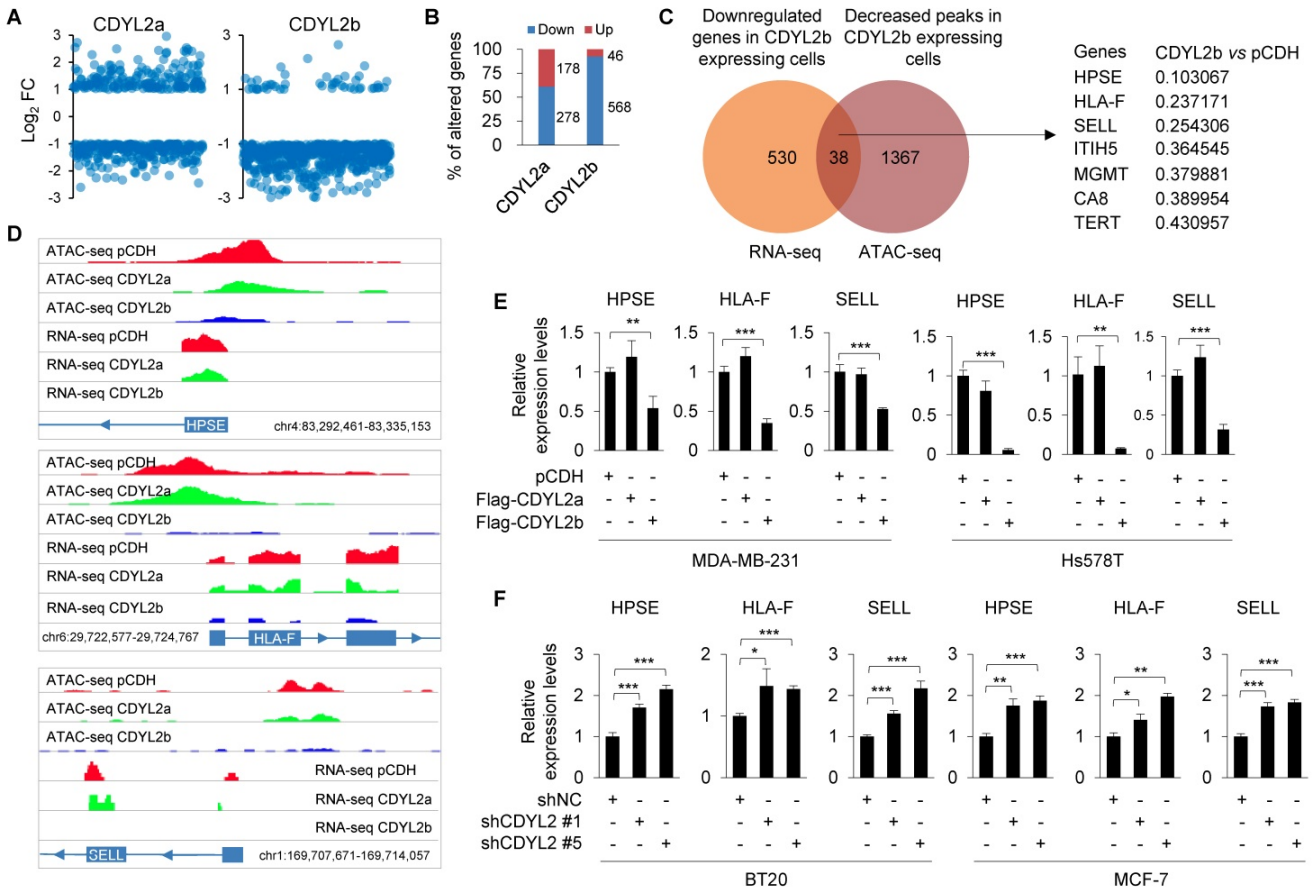
** CDYL2b transcriptionally represses HPSE, HLA-F, and SELL expression.** (A) Hs578T cells stably expressing pCDH, Flag-CDYL2a, and Flag-CDYL2b were subjected to RNA-seq analysis. The distribution of differentially expressed genes in CDYL2a or CDYL2b overexpressing cells is shown. (B) The percentage of upregulated or downregulated genes in CDYL2a or CDYL2b overexpressing H578T cells is shown. (C) Integrative analysis of RNA-seq and ATAC-seq results. Venn diagram showing overlapping genes (n = 38) between the downregulated genes from RNA-Seq and genes with decreased chromatin accessibility from ATAC-seq in CDYL2b overexpressing cells compared to pCDH expressing control cells. (D) RNA-seq and ATAC-seq reads around transcription starting sites of HPSE, HLA-F, and SELL genes are shown. (E) qPCR analysis of the expression levels of HPSE, HLA-F, and SELL in MDA-MB-231 and Hs578T cells stably expressing pCDH, Flag-CDYL2a, or Flag-CDYL2b. (F) qPCR analysis of the expression levels of HPSE, HLA-F, and SELL in BT20 and MCF-7 cells stably expressing shNC, shCDYL2 #1, or shCDYL2 #5. *, ** and ***, are significant differences at *p*<0.05, *p*<0.01, and *p*<0.001 levels, respectively.

As CDYL2b localizes to heterochromatin that plays important roles in transcriptional silencing, we investigated whether it suppresses breast cancer cell invasive and metastatic potential by transcriptional repression of target genes. To test this notion, we analyzed the above-generated RNA-seq data from Hs578T cells stably expressing pCDH, Flag-CDYL2a, or Flag-CDYL2b and focused on genes that were only affected by ectopic expression of CDYL2a or CDYL2b. Genes with FDR < 5% and log_2_ fold change (FC) > 1 or <-1 were considered differentially expressed. Results showed that 92.5% (568/614) and 61.0% (278/456) of the genes were downregulated in CDYL2b- and CDYL2a-expressing cells, respectively (**Figure 6A-B**). Gene ontology (GO) enrichment analyses revealed that CDYL2a regulating genes were involved in biological processes such as fat cell differentiation, cellular protein metabolism, inflammatory response, positive regulation of axonogenesis, and endothelial cell proliferation (**Figure S15A**). In contrast, genes regulated by CDYL2b were mainly involved in transcription regulation, cell migration, and cell adhesion (**Figure S15B**), supporting a role for CDYL2b in regulating gene transcription and cell migration.

Transcriptionally repressed genes are often organized within “closed” chromatin domains, marked by different histone modifications, such as H3K9me3. Given that CDYL2b co-localizes and interacts with H3K9me3 (**Figures 4A and S11A**), we surmised that CDYL2b may affect chromatin accessibility. To test this notion, Hs578T cells stably expressing pCDH, Flag-CDYL2a, or Flag-CDYL2b were subjected to ATAC-seq, a powerful method to examine genome-wide chromatin accessibility 36. We considered peaks with FDR < 5% and fold change > 2 or <0.5 as differentially accessible. ATAC-seq identified 1451 differentially accessible peaks in CDYL2b expressing cells compared to pCDH expressing control cells. Out of them, 96.8% (1405/1452) of the differentially accessible peaks were less accessible and 3.2% (47/1452) were more accessible in CDYL2b expressing cells, respectively. These results support a main role of CDYL2b as a repressor of transcription. Integrative analysis of ATAC-seq and RNA-seq data found that thirty-eight genes had significant reduced expression levels and decreased chromatin accessibility in cells expressing CDYL2b compared to those in pCDH- and CDYL2a- expressing cells (**Figure 6C**). According to the fold change of downregulated genes and decreased peaks in CDYL2b overexpressing cells compared to cells expressing pCDH, we chose three genes, heparanase (HPSE), major histocompatibility complex class I, F (HLA-F), and selectin L (SELL) for further validation (**Figure 6C-D**). qPCR analysis demonstrated that the expression levels of HPSE, HLA-F, and SELL were downregulated in both MDA-MB-231 and Hs578T cells expressing CDYL2b but not CDYL2a (**Figure 6E**). Depletion of CDYL2 by two independent shRNAs resulted in the upregulation of HPSE, HLA-F, and SELL in both BT20 and MCF-7 cells (**Figure 6F**). To further validate these results, we examined the expression levels of HPSE, HLA-F, and SELL in primary breast tumors with high CDYL2b expression (**Figure 1B**). Results showed that the expression levels of HPSE, HLA-F, and SELL were downregulated in most tumor tissues with high CDYL2b expression compared to the expression levels in matched normal breast tissues (**Figure S16A**). Consistently, reduced expression of HPSE, HLA-F, and SELL was also observed in CDYL2b-overexpressing xenograft tumors as compared with pCDH expressing controls (**Figure S16B**). Collectively, these results suggest that CDYL2b represses HPSE, HLA-F, and SELL expression.

Since CDYL2b may bind to H3K9me3, we hypothesized that CDYL2b may be recruited to the H3K9me3 containing promoter region of those three genes, resulting in transcription repression of their expression. To test this hypothesis, we analyzed the H3K9me3 enrichment regions at the promoters of these three genes at the UCSC website and designed four qPCR primers at the corresponding sites (**Figure S17A**). ChIP-qPCR was performed using Hs578T cells stably expressing pCDH, Flag-CDYL2a, or Flag-CDYL2b. Results showed that CDYL2b was enriched at least in one H3K9me3-containing region of these promoters. In contrast, CDYL2a was not enriched at these regions (**Figure S17B**). These experiments showed that CDYL2b might bind to the H3K9me3 region of HPSE, HLA-F, and SELL promoters, thus repressing their expression.

**Figure 7:**
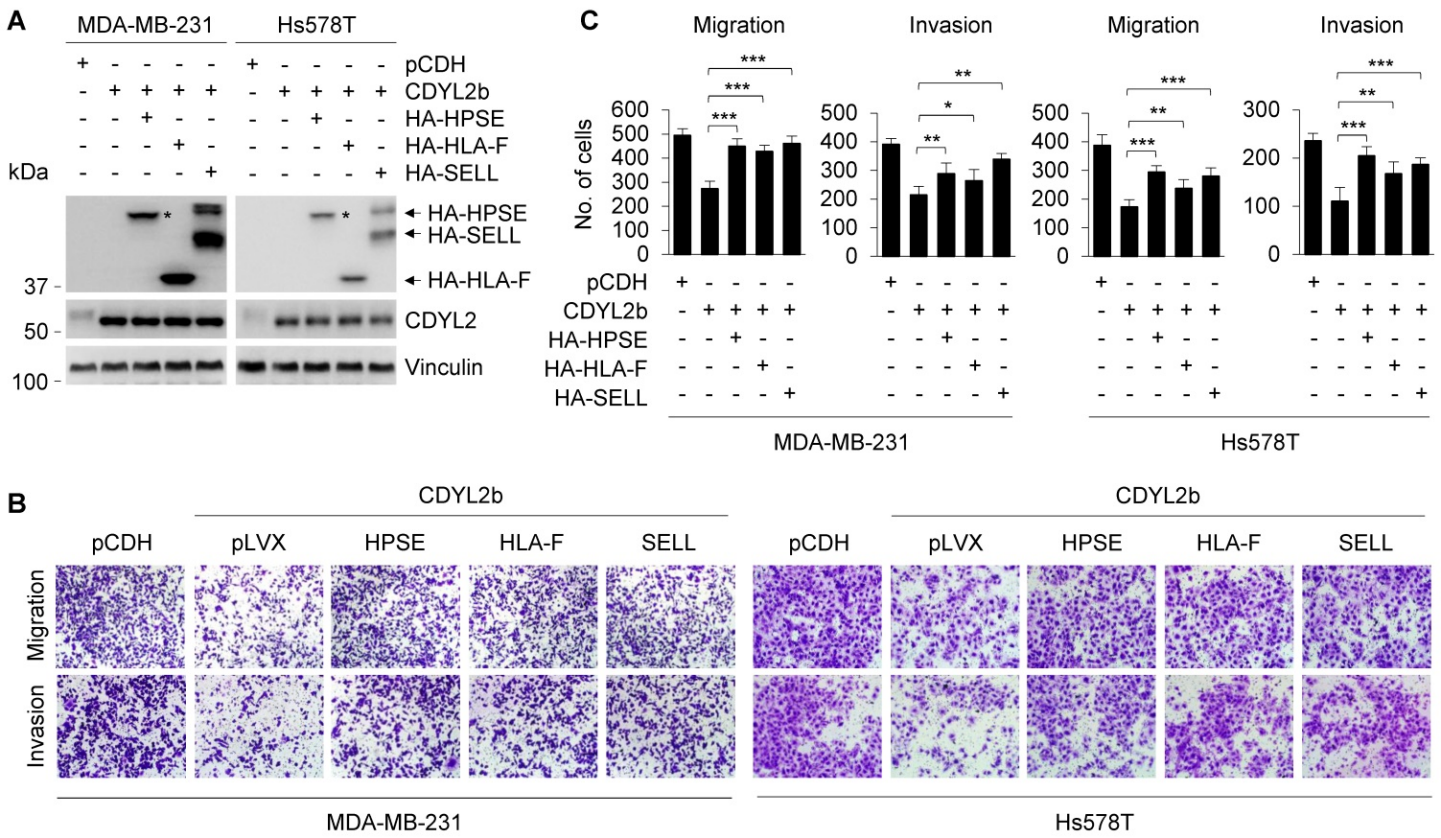
** CDYL2b inhibits breast cancer cell migration and invasion through transcriptional inhibition of HPSE, HLA-F, and SELL.** (A) MDA-MB-231 and Hs578T cells stably expressing pCDH, Flag-CDYL2b, or Flag-CDYL2b were transfected with plasmid DNAs encoding HA-HPSE, HA-HLA-F, or HA-SELL. The expression status of HA-HPSE, HA-HLA-F, or HA-SELL in these cells was validated by immunoblotting with an anti-HA antibody. (B-C) The established cell lines were subjected to Boyden's chamber migration assays and Matrigel-coated invasion assays. Representative images of Transwell migration and invasion assays are shown in B and corresponding quantitative results are shown in C. *, ** and *** are significant differences at *p*<0.05, *p*<0.01, and *p*<0.001 levels, respectively.

Previous studies have found that HPSE and SELL can promote the invasion and metastasis of cancer cells, and the expression of HLA-F is closely related to the invasion of cancer 56-58. To further verify that CDYL2b affects the migratory and invasive potential of breast cancer cells through transcriptional repression of HPSE, HLA-F, and SELL, we conducted rescue experiments by restoring HPSE, HLA-F, and SELL expression in MDA-MB-231 and Hs578T cells stably expressing CDYL2b (**Figure 7A**). Transwell cell migration and invasion assays showed that overexpression of CDYL2b in MDA-MB-231 and Hs578T cells suppressed breast cancer migration and invasion, which was partially rescued by expressing HPSE, HLA-F, and SELL in these cells (**Figure 7B-C**). These data confirmed once again that CDYL2b may affect the invasion and metastasis of breast cancer cells through, at least partly, transcriptional repression of HPSE, HLA-F, and SELL.

## Discussion

In this study, we report for the first time that two CDYL2 transcript variants, CDYL2a and CDYL2b, were differentially expressed in human breast cancer cell lines and primary breast tumors, and exerted discrete functional and mechanistic roles in breast cancer growth and metastasis through different molecular mechanisms.

Data indicate that spermatogenesis and carcinogenesis share a number of biological processes, such as cell survival, migration, and genome maintenance 32. Consequently, many of the transiently expressed genes in developing germ cells act as proto-oncogenes and oncogenes in various types of human cancers 32, 33. For instance, the fibrous sheath interacting protein 1 (FSIP1), a spermatogenesis related testicular antigen, is aberrantly expressed in breast cancer and regulates its growth and invasiveness 59. The sperm-associated antigen 1 (SPAG1) contributes to the early spread and poor prognosis of pancreatic adenocarcinoma 60. In addition, the spermatogenesis-associated protein 2 (SPATA2) has been shown to be a novel predictor of the outcome in ovarian cancer 61. The human genome encodes three CDY family proteins, including one on the Y chromosome (CDY) and two on autosomes (CDYL and CDYL2) 19. CDY is expressed exclusively in the testis 25 and has an important role in spermatogenesis 62. In addition, CDYL enables to regulate histone acetylation and crotonylation during spermatogenesis 27. Consequently, knockout of CDYL results in a severe progressive infertility in male mice due to the deficiency in spermatogonia maintenance 26. Recently, a gene expression profiling study identified CDYL as one of the fourteen differentially expressed genes between breast tumors with and without visceral metastatic disease 63. In addition, CDYL promotes the chemoresistance of small cell lung cancer by regulating H3K27me3 at the cyclin dependent kinase inhibitor 1C (CDKN1C) promoter 64. However, the functional and mechanistic role for CDYL2 in human cancer remains unexplored. In this study, we provided evidence that CDYL2 might generate four transcript variants (**Figure S6**). The most frequently and highly expressed CDYL2 variants in breast cancer cell lines and primary breast tumors were CDYL2a and CDYL2b. Especially, CDYL2a was upregulated in the majority of breast cancer cell lines and primary breast tumors, while CDYL2b was mainly expressed in luminal and HER2-positive ones (**Figure 1**). Functionally, CDYL2a played a protumorigenic role, whereas CDYL2b had an antimetastatic function in breast cancer progression (**Figure 2-3**).

The different subcellular locations of protein isoforms may contribute to its functional diversity. In our study, we found that CDYL2a partially co-localized with SC35, a well-established marker for nuclear speckles 46 (**Figure 4**). Nuclear speckles are nuclear domains enriched in pre-mRNA splicing factors and are involved in splicing regulation 45. Among these different modes of alternative splicing, exon skipping accounts for 40% of the entire alternative splicing events 65. Mechanistic investigations further revealed that CDYL2a was involved in regulating exon skipping events of its target genes, including FIP1L1, NKTR, and ADD3 (**Figure 5**). In support of our findings, an exon skipping event of FIP1L1 has been documented in colon cancer 66. NK-TR is specifically expressed in natural killer (NK) cells and is required for NK-like activity in human T cells 50. A recent study shows that the 5' region of the NK-TR mRNA has two sites of alternate splicing, and interleukin 2 (IL-2) regulates the expression of the NK-TR gene via an alternate RNA splicing mechanism 67. In addition, ADD3 alternative splicing events have been reported in HER2-positive breast cancer 53 and in non-small cell lung cancer 54. However, the detailed mechanism by which the alternative splicing of those three genes regulates breast cancer growth certainly deserves further investigation.

A recent study demonstrated that CDYL2 is mainly localized in the nucleus and interacts with H3K9me3 20. In our study, we found that the subcellular localization of CDYL2b was similar to the one of CDYL2, as reported previously 20. CDYL2b might bind to the H3K9me3 and transcriptionally repressed several metastasis-promoting genes, such as HPSE, HLA-F, and SELL (**Figure 6**). It has been reported that the HPSE levels in sera of patients with breast cancer are much higher than those in benign breast disease and healthy people 68 and HPSE may promote bone metastasis of breast cancer but not the tumor growth 69. In addition, microRNA-1258 can inhibit the expression and enzymatic activity of HPSE and thus suppress the brain metastasis of breast cancer 70. HLA-F is a member of the non-classical HLA class I molecules. The expression of HLA-F closely correlates with nodal involvement, lymphatic invasion, venous invasion, and poor prognosis in patients with gastric cancer 58. In gliomas, the expression of HLA-F also correlates with malignant phenotype and poor overall survival 71. In stage II breast cancer, overall survival of patients with HLA-F expression is lower than the one in those without HLA-F expression 72. SELL also participates in breast cancer metastasis. Depletion of SELL inhibits migration but not the proliferation of MDA-MB-231 cells 57. Hence, CDYL2b might regulate the migration, invasion, and metastasis of breast cancer through, at least partly, regulating HPSE, HLA-F, and SELL expression.

One unanswered question in this study is how CDYL2a and CDYL2b transcript variants are regulated during breast cancer progression. For alternative splicing, 99% of all exons are flanked by the intronic dinucleotides GT and AG at the 5' and 3' splice sites, respectively 73. Bioinformatic analyses revealed the absence of canonical (GT-AG) and non-canonical (GC-AG) splice sites in the exon-intron boundary at the first four exons of CDYL2 (data not shown), arguing against a role for splicing in their generation. Analysis of the promoter sequence of CDYL2a and CDYL2b using the UCSC Genome Browser database found that their promoter regions contain multiple transcription factor binding sites (data not shown), indicating that these transcription factors may alternatively use the distinct transcription start sites to induce CDYL2a and CDYL2b expression. However, the detailed mechanism underlying the generation of CDYL2a and CDYL2b in human breast cancer cell lines and breast tumors remains to be investigated in the near future.

## Conclusions

In summary, findings presented suggest that CDYL2 transcript variants exert discrete roles in breast cancer growth and metastasis through differentially regulating alternative splicing and transcription events. These findings suggest that the pharmacological inhibition of CDYL2a but not CDYL2b isoform would be an effective strategy for breast cancer therapy.

## Supplementary Material

Supplementary figures and tables.

## Abbreviations

ADD3: adducin 3
ATAC-seq: assay for transposase-accessible chromatin with high throughput sequencing
ATP5F1C: ATP synthase F1 subunit gamma
CDYL2): CDYL2, chromodomain Y-like 2
ChIP: chromatin immunoprecipitation
FIP1L1: factor interacting with PAPOLA and CPSF1
HLA-F: major histocompatibility complex class I, F
HPSE: heparanase
IP: immunoprecipitation
NKTR: natural killer cell triggering receptor
qPCR: quantitative real-time PCR
RNA-seq: RNA-sequencing
RT-PCR: reverse transcription-polymerase chain reaction
SELL: selectin L

## References

[1] Bray F, Ferlay J, Soerjomataram I, Siegel RL, Torre LA, Jemal A (2018). Global cancer statistics 2018: GLOBOCAN estimates of incidence and mortality worldwide for 36 cancers in 185 countries. CA Cancer J Clin.

[2] Perou CM, Sorlie T, Eisen MB, van de Rijn M, Jeffrey SS, Rees CA (2000). Molecular portraits of human breast tumours. Nature.

[3] Kast K, Link T, Friedrich K, Petzold A, Niedostatek A, Schoffer O (2015). Impact of breast cancer subtypes and patterns of metastasis on outcome. Breast Cancer Res Treat.

[4] Dai X, Li T, Bai Z, Yang Y, Liu X, Zhan J (2015). Breast cancer intrinsic subtype classification, clinical use and future trends. Am J Cancer Res.

[5] Stricker TP, Brown CD, Bandlamudi C, McNerney M, Kittler R, Montoya V (2017). Robust stratification of breast cancer subtypes using differential patterns of transcript isoform expression. PLoS Genet. 2017.

[6] Tyanova S, Albrechtsen R, Kronqvist P, Cox J, Mann M, Geiger T (2016). Proteomic maps of breast cancer subtypes. Nat Commun.

[7] de Klerk E, t Hoen PA (2015). Alternative mRNA transcription, processing, and translation: insights from RNA sequencing. Trends Genet.

[8] Davuluri RV, Suzuki Y, Sugano S, Plass C, Huang TH (2008). The functional consequences of alternative promoter use in mammalian genomes. Trends Genet.

[9] Qin Z, Stoilov P, Zhang X, Xing Y (2018). SEASTAR: systematic evaluation of alternative transcription start sites in RNA. Nucleic Acids Res.

[10] Wiesner T, Lee W, Obenauf AC, Ran L, Murali R, Zhang QF (2015). Alternative transcription initiation leads to expression of a novel ALK isoform in cancer. Nature.

[11] Oltean S, Bates DO (2014). Hallmarks of alternative splicing in cancer. Oncogene.

[12] Nilsen TW, Graveley BR (2010). Expansion of the eukaryotic proteome by alternative splicing. Nature.

[13] Wang ET, Sandberg R, Luo S, Khrebtukova I, Zhang L, Mayr C (2008). Alternative isoform regulation in human tissue transcriptomes. Nature.

[14] Elkon R, Ugalde AP, Agami R (2013). Alternative cleavage and polyadenylation: extent, regulation and function. Nat Rev Genet.

[15] Zhang H, Brown RL, Wei Y, Zhao P, Liu S, Liu X (2019). CD44 splice isoform switching determines breast cancer stem cell state. Genes Dev.

[16] Brown RL, Reinke LM, Damerow MS, Perez D, Chodosh LA, Yang J (2011). CD44 splice isoform switching in human and mouse epithelium is essential for epithelial-mesenchymal transition and breast cancer progression. J Clin Invest.

[17] Xu Y, Gao XD, Lee JH, Huang H, Tan H, Ahn J (2014). Cell type-restricted activity of hnRNPM promotes breast cancer metastasis via regulating alternative splicing. Genes Dev.

[18] Safikhani Z, Smirnov P, Thu KL, Silvester J, El-Hachem N, Quevedo R (2017). Gene isoforms as expression-based biomarkers predictive of drug response in vitro. Nat Commun.

[19] Dorus S, Gilbert SL, Forster ML, Barndt RJ, Lahn BT (2003). The CDY-related gene family: coordinated evolution in copy number, expression profile and protein sequence. Hum Mol Genet.

[20] Fischle W, Franz H, Jacobs SA, Allis CD, Khorasanizadeh S (2008). Specificity of the chromodomain Y chromosome family of chromodomains for lysine-methylated ARK(S/T) motifs. J Biol Chem.

[21] Cavalli G, Paro R (1998). Chromo-domain proteins: linking chromatin structure to epigenetic regulation. Curr Opin Cell Biol.

[22] Holden HM, Benning MM, Haller T, Gerlt JA (2001). The crotonase superfamily: divergently related enzymes that catalyze different reactions involving acyl coenzyme a thioesters. Acc Chem Res.

[23] Zhang J, Ibrahim MM, Sun M, Tang J (2015). Enoyl-coenzyme A hydratase in cancer. Clin Chim Acta.

[24] Yeh CS, Wang JY, Cheng TL, Juan CH, Wu CH, Lin SR (2006). Fatty acid metabolism pathway play an important role in carcinogenesis of human colorectal cancers by Microarray-Bioinformatics analysis. Cancer Lett.

[25] Lahn BT, Page DC (1999). Retroposition of autosomal mRNA yielded testis-specific gene family on human Y chromosome. Nat Genet.

[26] Xia X, Zhou X, Quan Y, Hu Y, Xing F, Li Z (2019). Germline deletion of Cdyl causes teratozoospermia and progressive infertility in male mice. Cell Death Dis.

[27] Liu S, Yu H, Liu Y, Liu X, Zhang Y, Bu C (2017). Chromodomain Protein CDYL Acts as a Crotonyl-CoA Hydratase to Regulate Histone Crotonylation and Spermatogenesis. Mol Cell.

[28] Kim ST, Sohn I, Do IG, Jang J, Kim SH, Jung IH (2014). Transcriptome analysis of CD133-positive stem cells and prognostic value of survivin in colorectal cancer. Cancer Genomics Proteomics.

[29] Balasubramanian D, Pearson JF, Kennedy MA (2019). Gene expression effects of lithium and valproic acid in a serotonergic cell line. Physiol Genomics.

[30] Yang SK, Hong M, Oh H, Low HQ, Jung S, Ahn S (2016). Identification of Loci at 1q21 and 16q23 That Affect Susceptibility to Inflammatory Bowel Disease in Koreans. Gastroenterology.

[31] Li L, Wang XQ, Liu XT, Guo R, Zhang RD (2019). Integrative analysis reveals key mRNAs and lncRNAs in monocytes of osteoporotic patients. Math Biosci Eng.

[32] Cheng YH, Wong EW, Cheng CY (2011). Cancer/testis (CT) antigens, carcinogenesis and spermatogenesis. Spermatogenesis.

[33] Wang C, Gu Y, Zhang K, Xie K, Zhu M, Dai N (2016). Systematic identification of genes with a cancer-testis expression pattern in 19 cancer types. Nat Commun.

[34] Livak KJ, Schmittgen TD (2001). Analysis of relative gene expression data using real-time quantitative PCR and the 2(-Delta Delta C(T)) Method. Methods.

[35] Li S, Hu Z, Zhao Y, Huang S, He X (2019). Transcriptome-Wide Analysis Reveals the Landscape of Aberrant Alternative Splicing Events in Liver Cancer. Hepatology.

[36] Buenrostro JD, Giresi PG, Zaba LC, Chang HY, Greenleaf WJ (2013). Transposition of native chromatin for fast and sensitive epigenomic profiling of open chromatin, DNA-binding proteins and nucleosome position. Nat Methods.

[37] Thul PJ, Lindskog C (2018). The human protein atlas: A spatial map of the human proteome. Protein Sci.

[38] Rhodes DR, Kalyana-Sundaram S, Mahavisno V, Varambally R, Yu J, Briggs BB (2007). Oncomine 3.0: genes, pathways, and networks in a collection of 18,000 cancer gene expression profiles. Neoplasia.

[39] Tang Z, Li C, Kang B, Gao G, Li C, Zhang Z (2017). GEPIA: a web server for cancer and normal gene expression profiling and interactive analyses. Nucleic Acids Res.

[40] Chandrashekar DS, Bashel B, Balasubramanya SAH, Creighton CJ, Ponce-Rodriguez I, Chakravarthi B (2017). UALCAN: A Portal for Facilitating Tumor Subgroup Gene Expression and Survival Analyses. Neoplasia.

[41] Edwards NJ, Oberti M, Thangudu RR, Cai S, McGarvey PB, Jacob S (2015). The CPTAC Data Portal: A Resource for Cancer Proteomics Research. J Proteome Res.

[42] Neve RM, Chin K, Fridlyand J, Yeh J, Baehner FL, Fevr T (2006). A collection of breast cancer cell lines for the study of functionally distinct cancer subtypes. Cancer Cell.

[43] Weigelt B, Peterse JL, van 't Veer LJ (2005). Breast cancer metastasis: markers and models. Nat Rev Cancer.

[44] Gyorffy B, Lanczky A, Eklund AC, Denkert C, Budczies J, Li Q (2010). An online survival analysis tool to rapidly assess the effect of 22,277 genes on breast cancer prognosis using microarray data of 1,809 patients. Breast Cancer Res Treat.

[45] Galganski L, Urbanek MO, Krzyzosiak WJ (2017). Nuclear speckles: molecular organization, biological function and role in disease. Nucleic Acids Res.

[46] Sleeman JE, Trinkle-Mulcahy L (2014). Nuclear bodies: new insights into assembly/dynamics and disease relevance. Curr Opin Cell Biol.

[47] Iwasaki J, Kondo T, Darmanin S, Ibata M, Onozawa M, Hashimoto D (2014). FIP1L1 presence in FIP1L1-RARA or FIP1L1-PDGFRA differentially contributes to the pathogenesis of distinct types of leukemia. Ann Hematol.

[48] Kaufmann I, Martin G, Friedlein A, Langen H, Keller W (2004). Human Fip1 is a subunit of CPSF that binds to U-rich RNA elements and stimulates poly(A) polymerase. EMBO J.

[49] Zhang L, Liu Y, Wang M, Wu Z, Li N, Zhang J (2017). EZH2-, CHD4-, and IDH-linked epigenetic perturbation and its association with survival in glioma patients. J Mol Cell Biol.

[50] Chambers CA, Gallinger S, Anderson SK, Giardina S, Ortaldo JR, Hozumi N (1994). Expression of the NK-TR gene is required for NK-like activity in human T cells. J Immunol.

[51] Deng B, Gong P, Li J, Cheng B, Ren W, Yang J (2013). Identification of the differentially expressed genes in SP2/0 myeloma cells from Balb/c mice infected with Trichinella spiralis. Vet Parasitol.

[52] Citterio L, Azzani T, Duga S, Bianchi G (1999). Genomic organization of the human gamma adducin gene. Biochem Biophys Res Commun.

[53] Eswaran J, Horvath A, Godbole S, Reddy SD, Mudvari P, Ohshiro K (2013). RNA sequencing of cancer reveals novel splicing alterations. Sci Rep.

[54] Langer W, Sohler F, Leder G, Beckmann G, Seidel H, Grone J (2010). Exon array analysis using re-defined probe sets results in reliable identification of alternatively spliced genes in non-small cell lung cancer. BMC Genomics.

[55] Rani SB, Rathod SS, Karthik S, Kaur N, Muzumdar D, Shiras AS (2013). MiR-145 functions as a tumor-suppressive RNA by targeting Sox9 and adducin 3 in human glioma cells. Neuro Oncol.

[56] Masola V, Zaza G, Gambaro G, Franchi M, Onisto M (2019). Role of heparanase in tumor progression: Molecular aspects and therapeutic options. Semin Cancer Biol.

[57] Chu JE, Xia Y, Chin-Yee B, Goodale D, Croker AK, Allan AL (2014). Lung-derived factors mediate breast cancer cell migration through CD44 receptor-ligand interactions in a novel ex vivo system for analysis of organ-specific soluble proteins. Neoplasia.

[58] Ishigami S, Arigami T, Okumura H, Uchikado Y, Kita Y, Kurahara H (2015). Human leukocyte antigen (HLA)-E and HLA-F expression in gastric cancer. Anticancer Res.

[59] Liu T, Zhang H, Sun L, Zhao D, Liu P, Yan M (2017). FSIP1 binds HER2 directly to regulate breast cancer growth and invasiveness. Proc Natl Acad Sci U S A.

[60] Neesse A, Gangeswaran R, Luettges J, Feakins R, Weeks ME, Lemoine NR (2007). Sperm-associated antigen 1 is expressed early in pancreatic tumorigenesis and promotes motility of cancer cells. Oncogene.

[61] Wieser V, Tsibulak I, Degasper C, Welponer H, Leitner K, Parson W (2019). Tumor necrosis factor receptor modulator spermatogenesis-associated protein 2 is a novel predictor of outcome in ovarian cancer. Cancer Sci.

[62] Kleiman SE, Yogev L, Hauser R, Botchan A, Bar-Shira Maymon B, Schreiber L (2003). Members of the CDY family have different expression patterns: CDY1 transcripts have the best correlation with complete spermatogenesis. Hum Genet.

[63] Savci-Heijink CD, Halfwerk H, Koster J, Horlings HM, van de Vijver MJ (2019). A specific gene expression signature for visceral organ metastasis in breast cancer. BMC Cancer.

[64] Qiu Z, Zhu W, Meng H, Tong L, Li X, Luo P (2019). CDYL promotes the chemoresistance of small cell lung cancer by regulating H3K27 trimethylation at the CDKN1C promoter. Theranostics.

[65] Liu S, Cheng C (2013). Alternative RNA splicing and cancer. Wiley Interdiscip Rev RNA.

[66] Huang X, Liu J, Mo X, Liu H, Wei C, Huang L (2019). Systematic profiling of alternative splicing events and splicing factors in left- and right-sided colon cancer. Aging.

[67] Rinfret A, Anderson SK (1993). IL-2 regulates the expression of the NK-TR gene via an alternate RNA splicing mechanism. Mol Immunol.

[68] Tang D, Piao Y, Zhao S, Mu X, Li S, Ma W (2014). Expression and correlation of matrix metalloproteinase-9 and heparanase in patients with breast cancer. Mol Oncol.

[69] Kelly T, Suva LJ, Huang Y, Macleod V, Miao HQ, Walker RC (2005). Expression of heparanase by primary breast tumors promotes bone resorption in the absence of detectable bone metastases. Cancer Res.

[70] Zhang L, Sullivan PS, Goodman JC, Gunaratne PH, Marchetti D (2011). MicroRNA-1258 suppresses breast cancer brain metastasis by targeting heparanase. Cancer Res.

[71] Feng E, Liang T, Wang X, Du J, Tang K, Wang X (2019). Correlation of alteration of HLA-F expression and clinical characterization in 593 brain glioma samples. J Neuroinflammation.

[72] Harada A, Ishigami S, Kijima Y, Nakajo A, Arigami T, Kurahara H (2015). Clinical implication of human leukocyte antigen (HLA)-F expression in breast cancer. Pathol Int.

[73] Venables JP (2004). Aberrant and alternative splicing in cancer. Cancer Res.

