# Supplementary material for: Discrete functional and mechanistic roles of chromodomain Y-like 2 (CDYL2) transcript variants in breast cancer growth and metastasis
Source: Theranostics. 2020 Apr 6;10(12):5242–58. doi: 10.7150/thno.43744 (PMC7196301; doi:10.7150/thno.43744)
Supplement: Supplementary file 1 — Supplementary figures and tables. [file thnov10p5242s1.pdf]

Supplementary Information for

Yang et al. Discrete functional and mechanistic roles of CDYL2 transcript variants in breast cancer growth and metastasis

The Supporting information contains:

Figures S1-S17

Tables S1-S5

Supplementary Figures and Figure legends

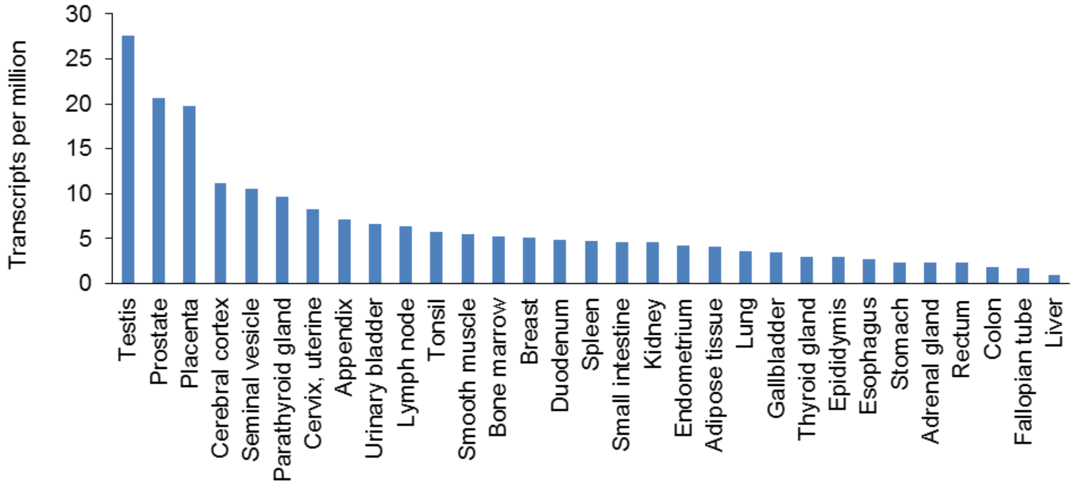

Figure S1. CDYL2 expression profiles in human normal tissues

The RNA expression profiles of CDYL2 in normal human tissues. Data was obtained from HPA database (https://www.proteinatlas.org/).

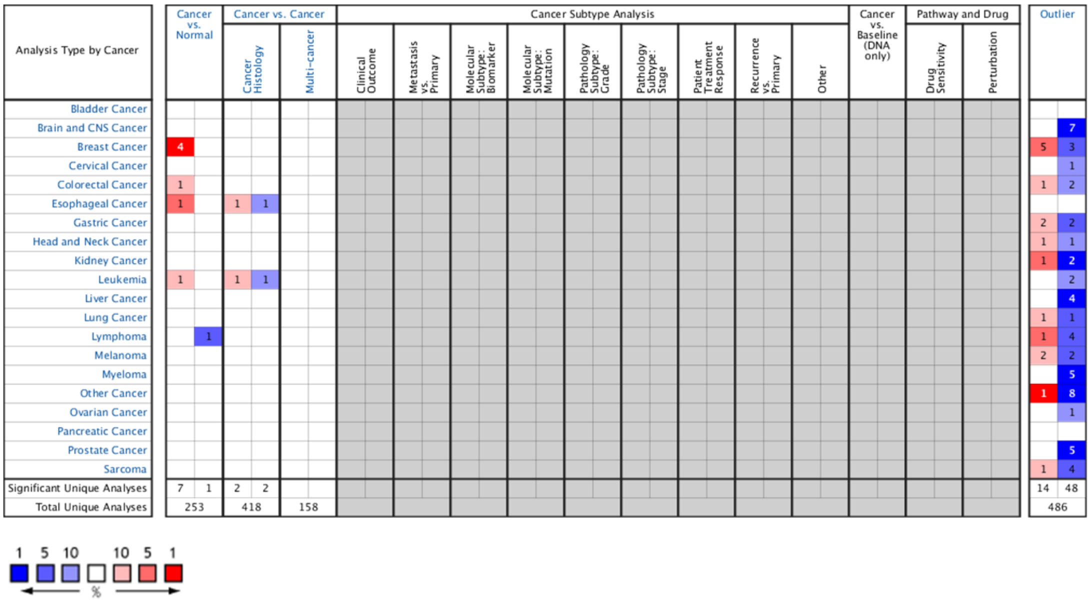

Figure S2. An overview of CDYL2 mRNA levels in a variety of human cancer according to Oncomine database

This graphic showed the numbers of datasets with statistically significant mRNA overexpression (red) or underexpression (blue) of CDYL2 gene (cancer vs. normal tissue). Cell color was determined by the best gene rank percentile for the analysis within the cells. The threshold was set as gene rank percentile (top 10%), *p* <0.01, and fold change>2.

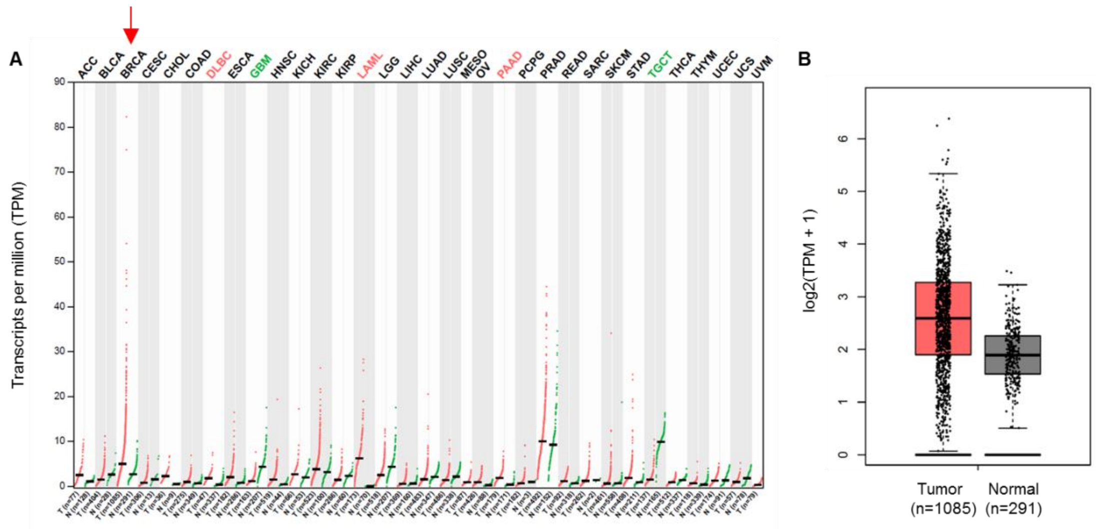

Figure S3. Analysis of CDYL2 mRNA levels in human tumors and normal issues using GEPIA database

(A) The CDYL2 mRNA expression profile across all tumor (T) samples and paired normal (N) tissues. The data was generated using GEPIA database. The RNA-seq results are reported as number of transcripts per million (TPM). Each dot represents expression of sample. The red arrow indicates breast invasive carcinoma (BRCA).

Abbreviations for tumor types: ACC, adrenocortical carcinoma; BLCA, bladder Urothelial Carcinoma; BRCA, breast invasive carcinoma; CESC, cervical squamous cell carcinoma and endocervical adenocarcinoma; CHOL, cholangio carcinoma; COAD, colon adenocarcinoma; DLBC, lymphoid neoplasm diffuse large B-cell lymphoma; ESCA, esophageal carcinoma; GBM, glioblastoma multiforme; HNSC, head and neck squamous cell carcinoma; KICH, kidney chromophobe; KIRC, kidney renal clear cell carcinoma; KIRP, kidney renal papillary cell carcinoma; LAML, acute myeloid leukemia; LGG, brain lower grade glioma; LIHC, liver hepatocellular carcinoma; LUAD, lung adenocarcinoma; LUSC, lung squamous cell carcinoma; MESO, mesothelioma; OV, ovarian serous cystadenocarcinoma; PAAD, pancreatic adenocarcinoma; PCPG, pheochromocytoma and paraganglioma; PRAD, prostate adenocarcinoma; READ, rectum adenocarcinoma; SARC, sarcoma; SKCM,skin cutaneous melanoma; STAD, stomach adenocarcinoma; TGCT, testicular germ cell

tumors; THCA, thyroid carcinoma; THYM, thymoma; UCEC, uterine oorpus endometrial carcinoma; UCS, uterine carcinosarcoma; UVM, uveal Melanoma.

(B) CDYL2 was up-regulated in human breast tumors. GEPIA was used for the analysis of CDYL2 expression, and the boxplot was plotted. The red and gray boxes represent breast tumors and normal breast tissues, respectively. The height of bar represents the median expression of certain tumor type or normal tissue.

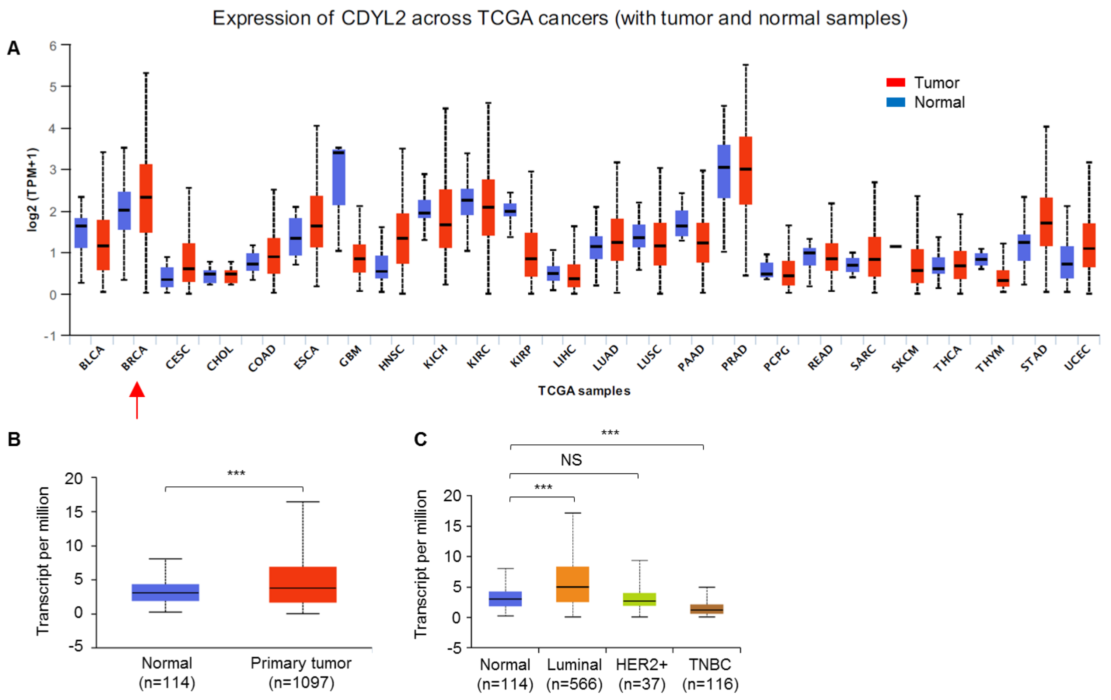

Figure S4. Analysis of CDYL2 mRNA levels using TCGA database

(A) Expression of CDYL2 across TCGA cancers with tumors and normal tissue samples. The red arrow indicates breast invasive carcinoma (BRCA).

Abbreviations for tumor types: BLCA, bladder Urothelial Carcinoma; BRCA, breast invasive carcinoma; CESC, cervical squamous cell carcinoma and endocervical adenocarcinoma; CHOL, cholangio carcinoma; COAD, colon adenocarcinoma; DLBC, lymphoid neoplasm diffuse large B-cell lymphoma; ESCA, esophageal carcinoma; GBM, glioblastoma multiforme; HNSC, head and neck squamous cell carcinoma; KICH, kidney chromophobe; KIRC, kidney renal clear cell carcinoma; KIRP, kidney renal papillary cell carcinoma; LIHC, liver hepatocellular carcinoma; LUAD, lung adenocarcinoma; LUSC, lung squamous cell carcinoma; PAAD, pancreatic adenocarcinoma; PCPG, pheochromocytoma and paraganglioma; PRAD, prostate adenocarcinoma; READ, rectum adenocarcinoma; SARC, sarcoma; SKCM, skin cutaneous melanoma; STAD, stomach adenocarcinoma; THCA, thyroid carcinoma; THYM, thymoma; UCEC, uterine oorpus endometrial carcinoma.

(B) Relative expression levels of CDYL2 in breast tumor tissues and normal tissues in TCGA.

(C) Relative expression levels of CDYL2 in different subtypes of breast tumors in TCGA. NS, no significance; ***p<0.001.

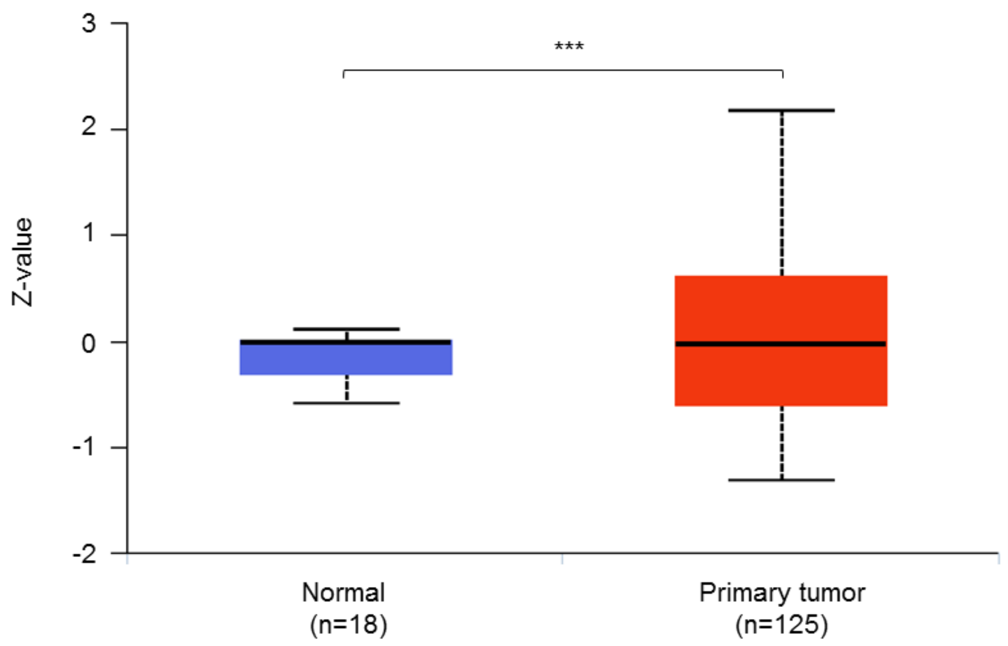

Figure S5. Analysis of CDYL2 protein expression levels in breast tumors and normal breast tissues using CPTAC database

Z-values represent standard deviations from the median across samples. Log2 Spectral count ratio values from CPTAC were first normalized within each sample profile and then normalized across samples. ***$p < 0.001$.

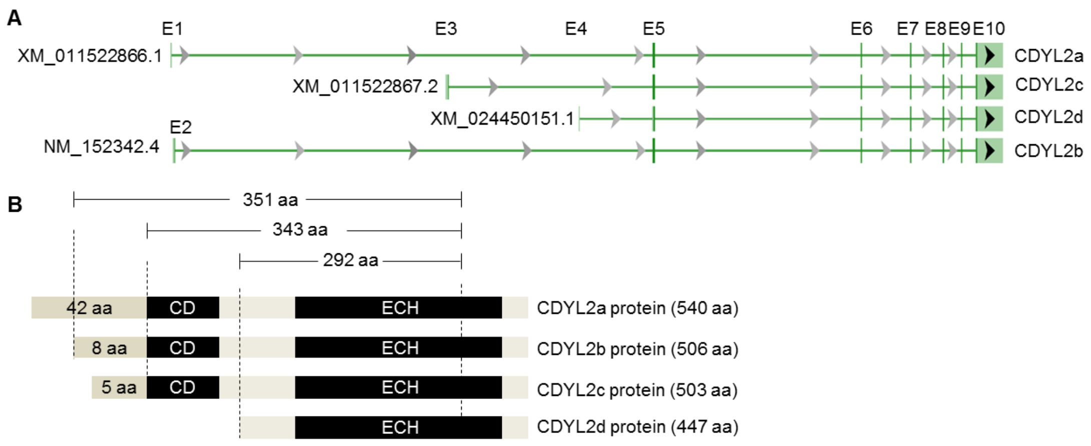

Figure S6. Human CDYL2 pre-mRNA generates four transcript variants

(A) Schematic representation of four CDYL2 transcription variants.

(B) Schematic representation of four CDYL2 protein isoforms.

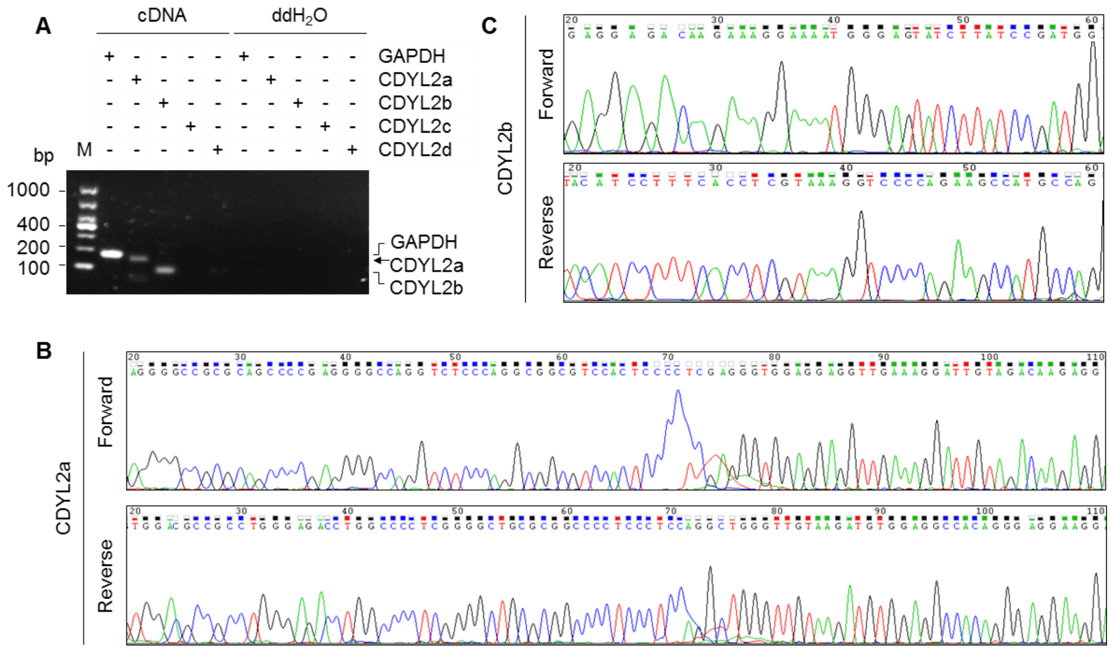

Figure S7. CDYL2a and CDYL2b are the predominant isoforms expressed in MCF-7 breast cancer cell line

(A) RT-PCR analysis of the expression levels of four CDYL2 variants in MCF-7 cells. GAPDH was used as a positive control.

(B-C) Sequencing of the PCR products from A.

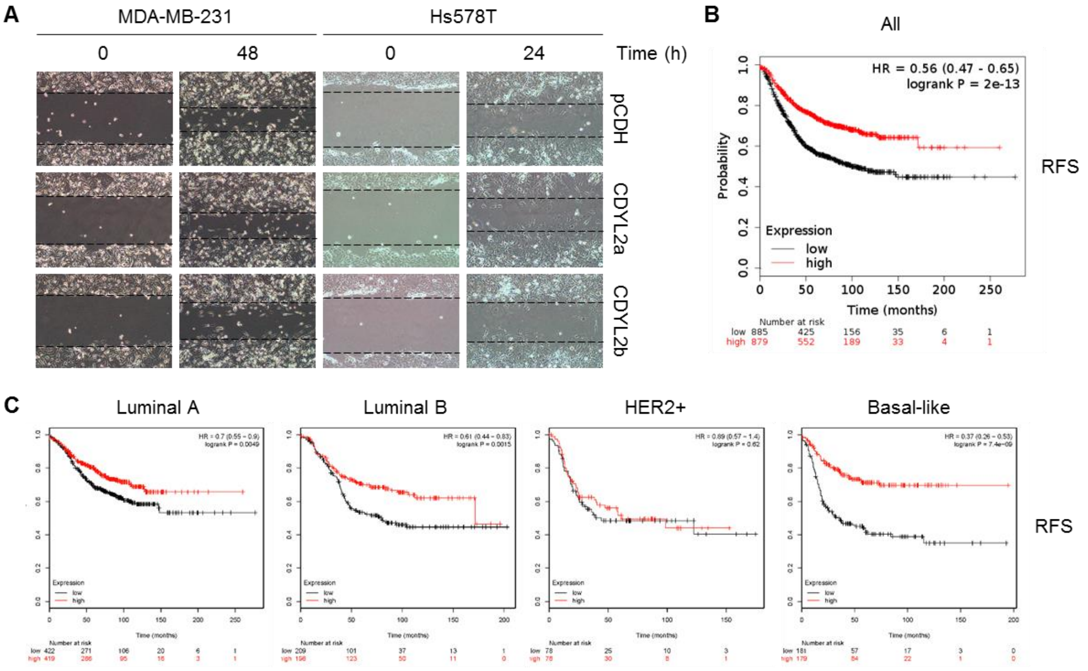

Figure S8. CDYL2b suppresses breast cancer cell migratory potential and its expression levels are negatively associated with prognosis of patients with breast cancer

(A) MDA-MB-231 and Hs578T cells stably expressing pCDH, Flag-CDYL2a, or Flag-CDYL2b were subjected to wound-healing assays. The corresponding quantitative results are shown in Fig. 2H.

(B-C) Kaplan-Meier curves of relapse-free survival (RFS) of breast cancer patients with high or low CDYL2 expression in Kaplan-Meier plotter database.

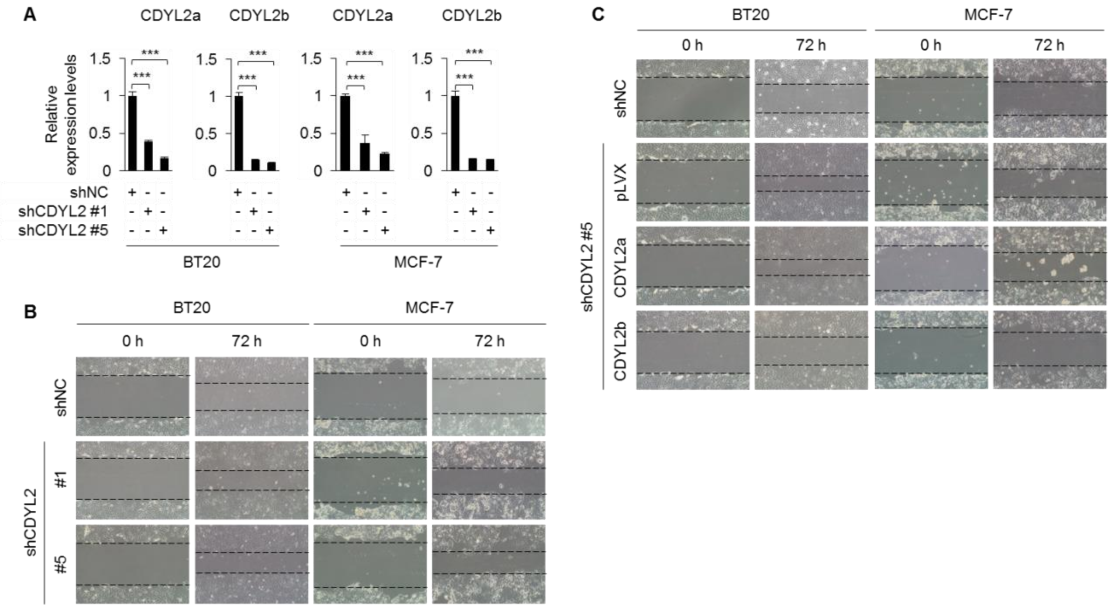

Figure S9. CDYL2 suppresses breast cancer cell migratory potential

(A) qPCR analysis of CDYL2 expression in BT20 and MCF-7 cells stably expressing shNC, shCDYL2 #1, or shCDYL2 #5. ***$p<0.001$.

(B) BT20 and MCF-7 cells stably expressing shNC, shCDYL2 #1, or shCDYL2 #5 were subjected to wound-healing assays. The corresponding quantitative results are shown in Fig. 3I.

(C) CDYL2-depleted BT20 and MCF-7 cells were reconstituted with Flag-CDYL2a and Flag-CDYL2b and then subjected to wound-healing assays. The corresponding quantitative results are shown in Fig. 3L.

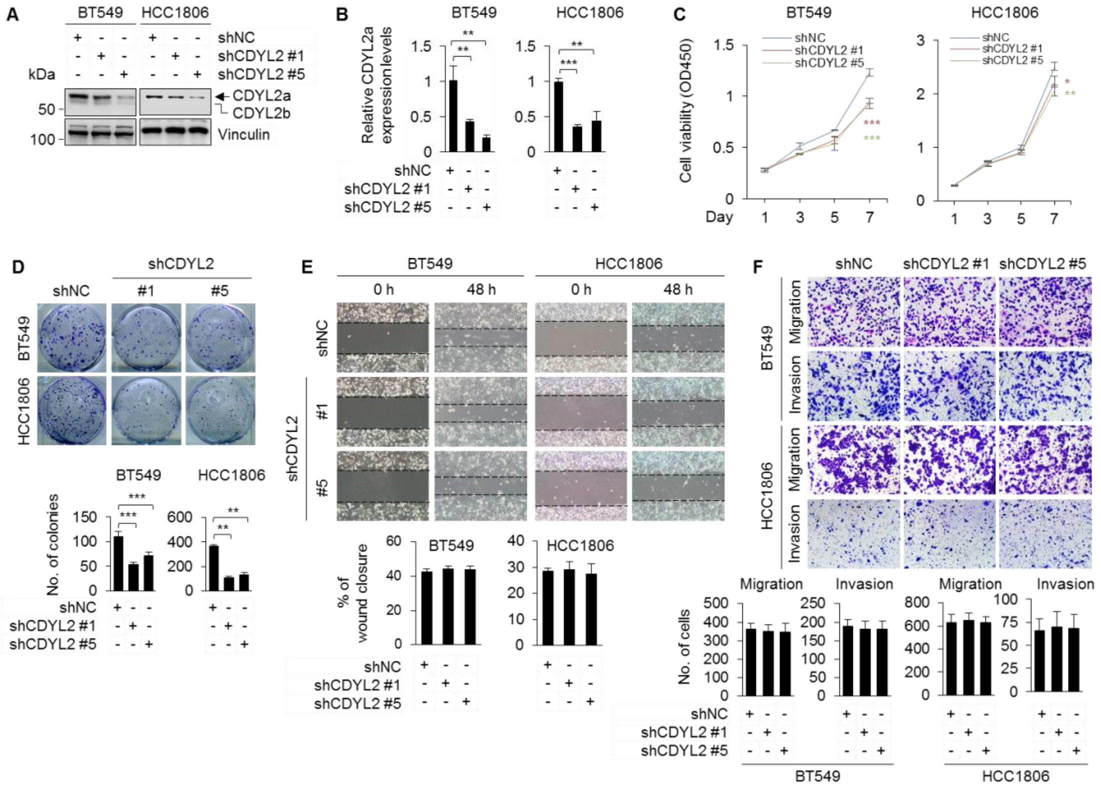

Figure S10. CDYL2a promotes breast cancer cell proliferation and colony formation

(A-B) Immunoblotting (A) and qPCR (B) analysis of CDYL2a expression in BT549 and HCC1806 cells stably expressing shNC, shCDYL2 #1, or shCDYL2 #5.

(C-F) BT549 and HCC1806 cells stably expressing shNC, shCDYL2#1, or shCDYL2#5 were subjected to cell proliferation assays using CCK-8 kit (C), colony formation assays (D), wound-healing assays (E), Boyden's chamber migration assays, and Matrigel-coated invasion assays (F). **p*<0.05; ***p*< 0.01; ****p*< 0.001.

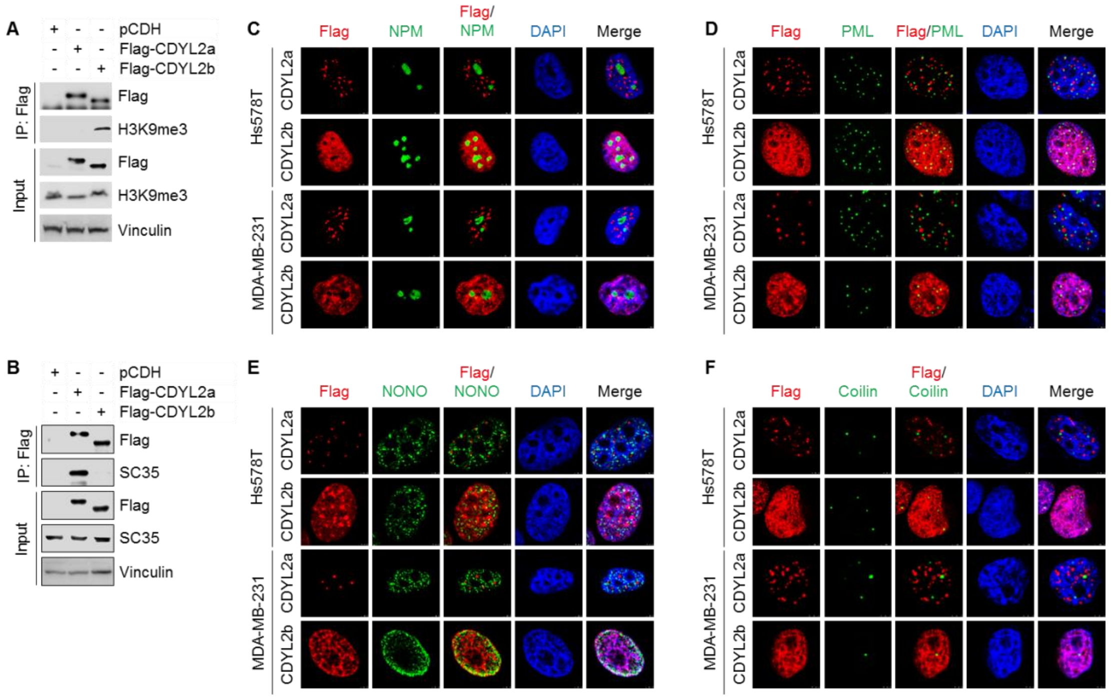

Figure S11. Subcellular localization of CDYL2a and CDYL2b

(A-B) HEK293T cells were transfected with pCDH, Flag-CDYL2a, or Flag-CDYL2b. After 48 h of transfection, cells were subjected to sequential IP and immunoblotting analyses with the indicated antibodies.

(C-F) MDA-MB-231 and Hs578T cells stably expressing pCDH, Flag-CDYL2a, or Flag-CDYL2b were stained with Flag (red), nucleophosmin (NPM) (C), promyelocytic leukemia (PML) (D), non-POU domain-containing octamer-binding protein (NONO) (E), and Coilin (F) by immunofluorescence. DNA was counterstained with DAPI.

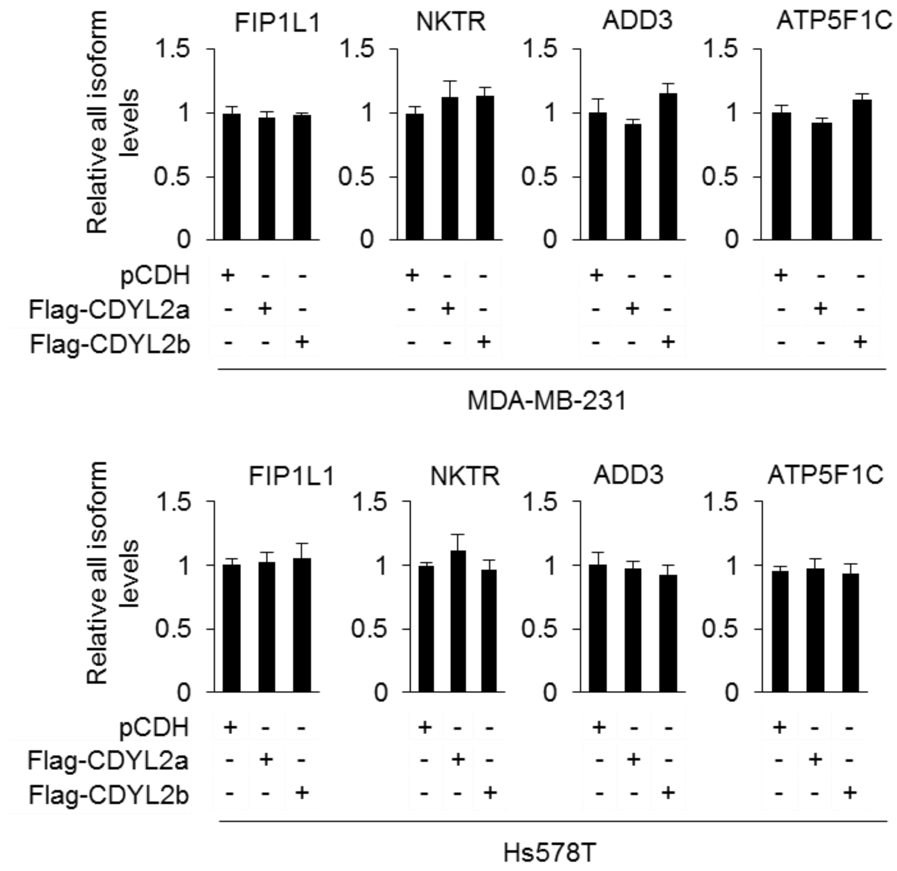

Figure S12. qPCR analysis of all isoform levels of FIP1L1, NKTR, ADD3, and ATP5FC1 genes in MDA-MB-231 and Hs578T cells stably expressing pCDH, Flag-CDYL2a, or Flag-CDYL2b

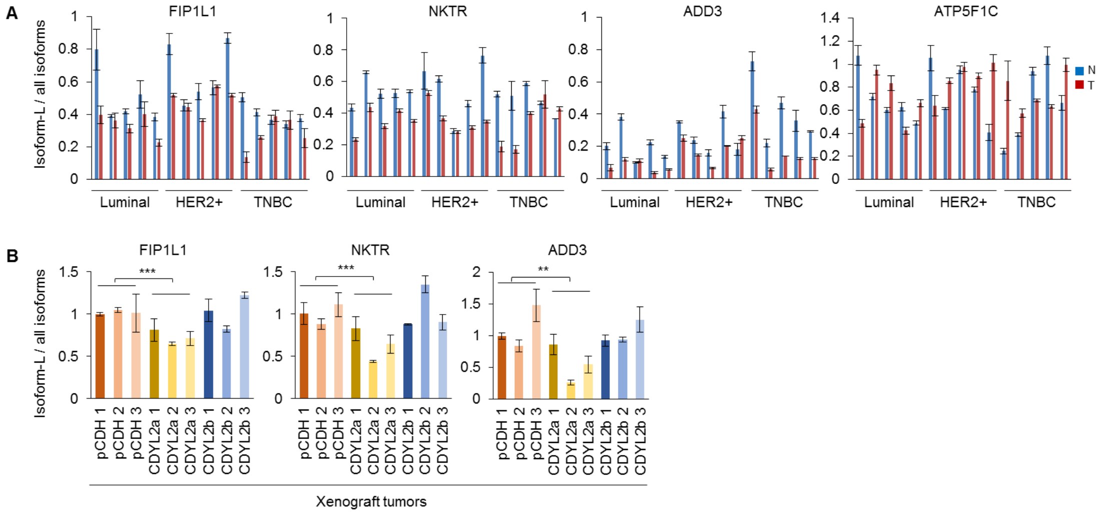

Figure S13. CDYL2a regulates alternative splicing of FIP1L1, NKTR, and ADD3 genes

(A) qPCR analysis was carried out with isoform-specific primers using 15 pairs of matched primary breast tumors and normal breast tissues. The ratio of the L isoforms to all isoforms of FIP1L1, NKTR, ADD3, and ATP5FC1 genes is shown.

(B) qPCR analysis was carried out with isoform-specific primers using tumor samples from xenograft tumor models shown in Fig.2E. The ratio of the L isoforms to all isoforms of FIP1L1, NKTR, and ADD3 genes is shown. **p< 0.01; ***p< 0.001.

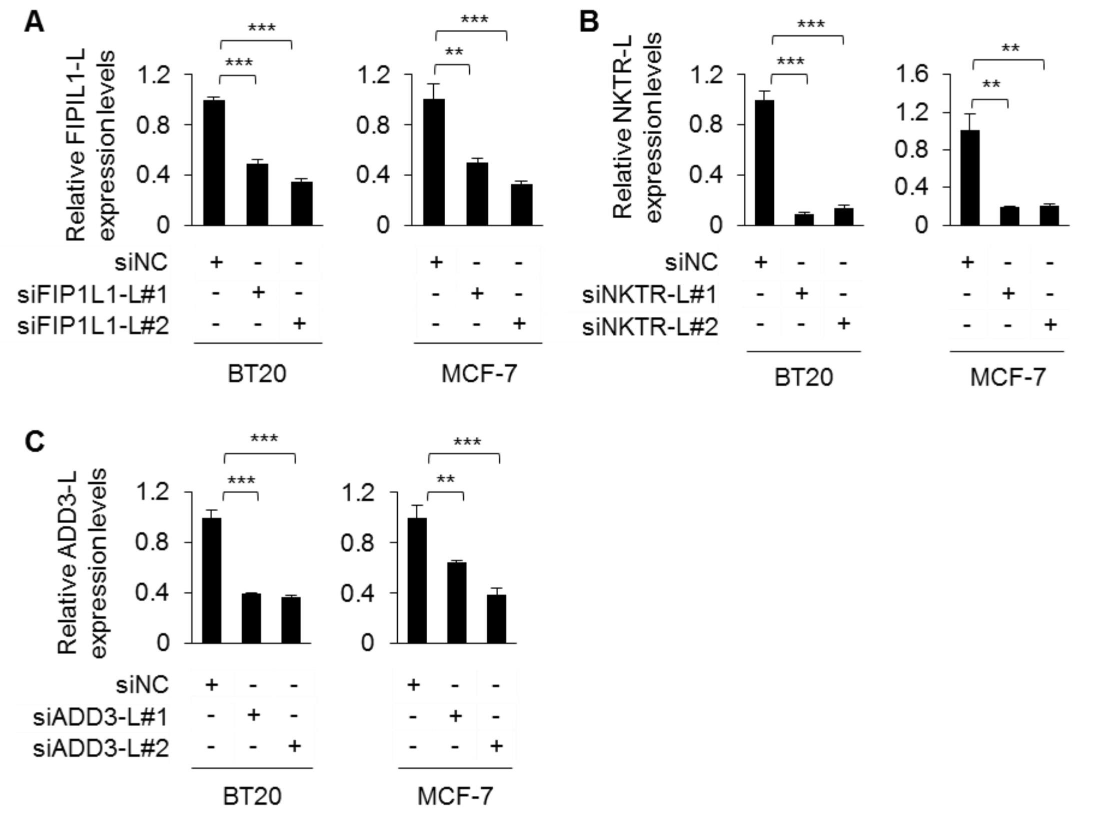

Figure S14. qPCR analysis of the L isoform expression levels of FIP1L1, NKTR, and ADD3 genes in BT20 and MCF-7 cells that were transfected with indicated siRNAs. **p<0.01; ***p< 0.001.

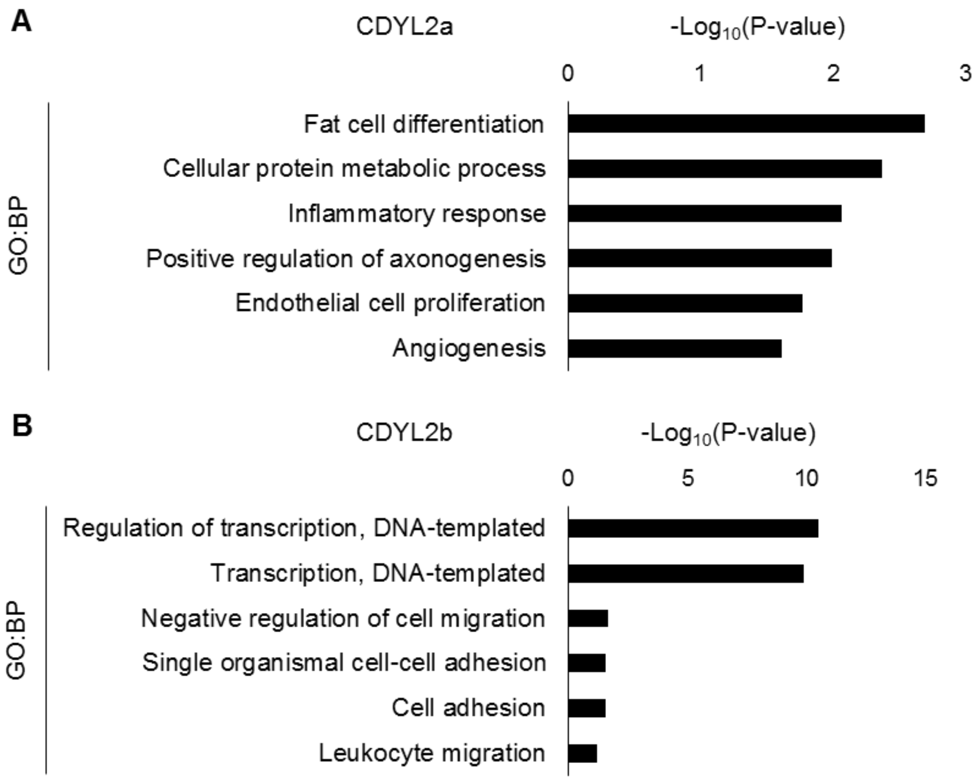

Figure S15. Gene ontology (GO) enrichment analysis of altered genes only in CDYL2a (A) or CDYL2b (B) overexpressing cells

BP, biological process

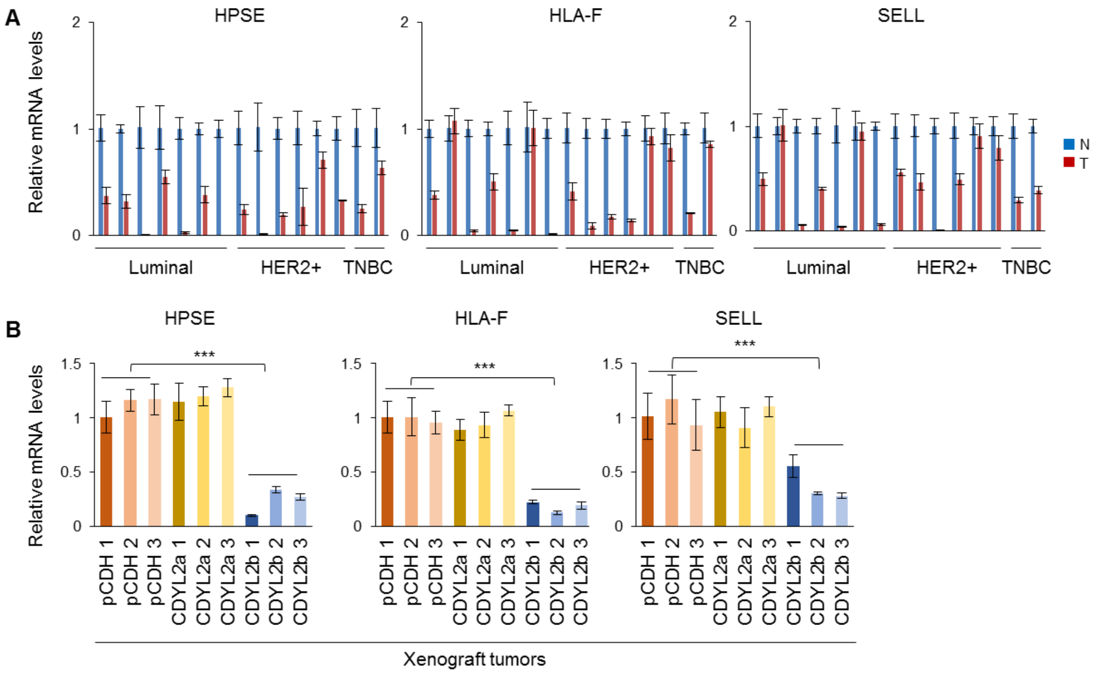

Figure S16. CDYL2b represses HPSE, HLA-F, and SELL expression

(A) qPCR analysis of HPSE, HLA-F, and SELL expression in 15 pairs of matched primary breast tumors and normal breast tissues. The expression levels of HPSE, HLA-F, or SELL in normal tissues were used as a control after normalization to the GAPDH levels.

(B) qPCR analysis of HPSE, HLA-F, and SELL expression in xenograft tumor samples from xenograft tumor models shown in Fig.2E. ***$p < 0.001$.

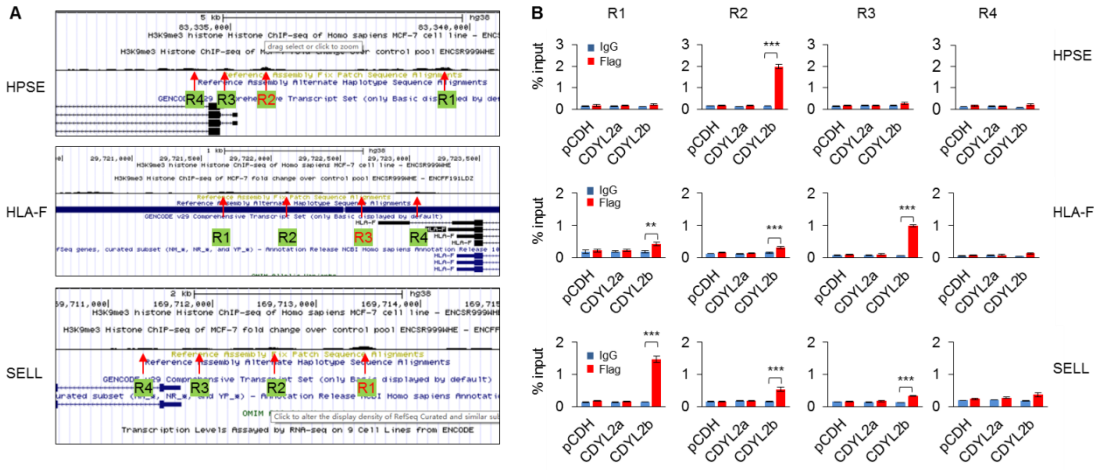

Figure S17. CDYL2b is enriched in the promoter regions of HPSE, HLA-F, and SELL

(A) The H3K9me3 enrichment region at HPSE, HLA-F, and SELL promoters in UCSC database.

(B) ChIP-qPCR analysis of the enrichment of CDYL2a or CDYL2b on the promoter regions of HPSE, HLA-F, and SELL genes in the presence of high H3K9me3 levels. **p< 0.01; ***p<0.001.

Supplementary Tables

Table S1. Information for expression vectors used in this study

Plasmids	Sources	Vectors
pCDH-Flag-CDYL2a	Subcloned	pCDH-CMV-MCS-EF1-Puro
pCDH-Flag-CDYL2b	Subcloned	pCDH-CMV-MCS-EF1-Puro
pLVX-Flag-CDYL2a	Subcloned	pLVX-IRES-Neo
pLVX-Flag-CDYL2b	Subcloned	pLVX-IRES-Neo
pLVX-HA-HPSE	Subcloned	pLVX-IRES-Neo
pLVX-HA-HLA-F	Subcloned	pLVX-IRES-Neo
pLVX-HA-SELL	Subcloned	pLVX-IRES-Neo
shCDYL2#1	Subcloned	pLKO.1-TRC
shCDYL2#5	Subcloned	pLKO.1-TRC
CDYL2b	Vigene Bioscience (#CH869225)	pEnter
HPSE	Vigene Bioscience (#CH806645)	pEnter
HLA-F	Vigene Bioscience (#CH864022)	pEnter
SELL	Vigene Bioscience (#CH832492)	pEnter

Table S2. Primers used for molecular cloning of expression vectors

Expression vectors	Primers	Sequences
shCDYL2#1	Forward	CCGGGGATTGTAGACAAGAGGAACTCGAGTTC CTCTTGTCTACAATCCTTTTTG
	Reverse	AATTCAAAAAGGATTGTAGACAAGAGGAACTCG AGTTCCTCTTGTCTACAATCC
shCDYL2#5	Forward	CCGGGGAAGCTGGAAGCGGAGAACTCGAGTT CTCCGCTTCCAGCTTCCTTTTTG
	Reverse	AATTCAAAAAGGAAGCTGGAAGCGGAGAACTC GAGTTCTCCGCTTCCAGCTTCC
pCDH-Flag-CDYL2a	Forward	GATTACAAGGATGACGACGATAAGATGGGCCAT TTCATCCTTCC
	Forward middle	GAGGTTGAAAGGATTGTAGACAAG
	Reverse	ATCCTTCGCGGCCGCGGATCCTCAGACTTCATA AATTTTGTCCTGCAGG
pCDH-Flag-CDYL2b	Forward	GATTACAAGGATGACGACGATAAGATGGCTTCT GGGGACCTTTAC
	Reverse	ATCCTTCGCGGCCGCGGATCCTCAGACTTCATA AATTTTGTCCTGCAGG
pLVX-Flag-CDYL2a	Forward	GATTACAAGGATGACGACGATAAGATGGGCCAT TTCATCCTTCC
	Reverse	ATCCTTCGCGGCCGCGGATCCTCAGACTTCATA AATTTTGTCCTGCAGG
pLVX-Flag-CDYL2b	Forward	GATTACAAGGATGACGACGATAAGATGGCTTCT GGGGACCTTTAC
	Reverse	ATCCTTCGCGGCCGCGGATCCTCAGACTTCATA AATTTTGTCCTGCAGG
pLVX-HA-HPSE	Forward	CGGAATTCGCCACCATGCTGCTGCGCTCGAAG
	Reverse	CTGGGACGTCGTATGGGTATCCGATGCAAGCA

		GCAACTTTGG
pLVX-HA-HLA-F	Forward	CGGAATTCGCCACCATGGCGCCCCGAAGC
	Reverse	CTGGGACGTCGTATGGGTATCCCACTGCAGCC TGAGAGTAGCTC
pLVX-HA-SELL	Forward	CGGAATTCGCCACCATGATATTTCCATGGAAATG TCAGAG
	Reverse	CTGGGACGTCGTATGGGTATCCATATGGGTCAT TCATACTTCTCTTGG
pLVX-CDYL2 samesence mutation	Forward	GTCCAGTGAAGAGAAAATTAGAGGCTGAAAAG GAC
	Reverse	GACGTAGTCCTTTTCAGCCTCTAATTTTCTCTTC A

Table S3. The sequences of siRNAs used in this study

siRNAs	Target sequences (5’-3’)
siFIP1L1#1	GTATTCCAATAACTGTACC
siFIP1L1#2	TCCAATAACTGTACCACCT
siNKTR #1	GAATGTGGTCTTTTGCAAA
siNKTR #2	GAGAATGTGGTCTTTTGCA
siADD3 #1	GCTTCATCTGTTTCACAAA
siADD3 #2	CAAACTCAGTCACCGCAAA
siADD3 #3	CGCAAAATGTCCCTGAAAA

Table S4. Primers for qPCR, conventional RT-PCR, and ChIP-qPCR analysis

Genes	Primers	Sequences
CDYL2a	Forward	ATTTCATCCTTCCTCCCTGTG
	Reverse	GTTCTTCCTCTTGTCTACAATCCT
CDYL2b	Forward	CCTGGCATGGCTTCTGG
	Reverse	CCATCGGATAAGATACTCCCATTT
CDYL2c	Forward	TGGGAAGGTGACCCTTAGT
	Reverse	CCATCGGATAAGATACTCCCATTT
CDYL2d	Forward	AGGAGGCCACAGTCTAGTT
	Reverse	AGCCTTTCCATCGGATAAGATAC
HPSE	Forward	TCCTGCGTACCTGAGGTTTG
	Reverse	CCATTCCAACCGTAACTTCTCCT
HLA-F	Forward	TGGCCCTGACCGATACTTG
	Reverse	GCAGGAATTGCGTGTCGTC
SELL	Forward	ACCCAGAGGGACTTATGGAAC
	Reverse	GCAGAATCTTCTAGCCCTTTGC
FIP1L1	Forward	CCAATAACTGTACCACCTCCAG
exon-specific	Reverse	CACGTGCAGAACGACTATCA
FIP1L1	Forward	GAGAACAGCAACATACAGGTCC
all isoforms	Reverse	GAATCAGAGGTGGAGCAGTGC
FIP1L1	Forward	GAGAACAGCAACATACAGGTCC
trans exon	Reverse	CACGTGCAGAACGACTATCA
NKTR	Forward	GCTTGTGCTCAGGAGAGAAA
exon-specific	Reverse	TGCAAAAGACCACATTCTCTT
NKTR	Forward	GCTTGTGCTCAGGAGAGAAA
all isoforms	Reverse	CCACACGATGGAACGTAGAA
NKTR	Forward	GCTTGTGCTCAGGAGAGAAA

trans exon	Reverse	CTTCAAAACCAGAAATAACCAGT
ADD3	Forward	TTCAGTCTCAAACTCAGTCACC
exon-specific	Reverse	AGACTTAATAGTAATCTCGATGTTTTCTA
ADD3	Forward	GGTAGTAAATGGCAAGGATGATAT
all isoforms	Reverse	AGACTTAATAGTAATCTCGATGTTTTCTA
ADD3	Forward	TCAAATACATGGCACAGAGGC
trans exon	Reverse	TTACTACCATGACAGGCACTTC
ATP5F1C	Forward	GTGCTGACAGCATGAGTATCTATGAC
exon-specific	Reverse	GAGGATGGAACTTGATTTTCATTAA
ATP5F1C	Forward	GTGCTGACAGCATGAGTATCTATGAC
all isoforms	Reverse	GCTGCAGCACCAGAGATAA
ATP5F1C	Forward	GTGCTGACAGCATGAGTATCTATGAC
trans exon	Reverse	CTTCGGACAAAGGCAGCAGTA
HPSE ChIP 1	Forward	GCTCAGCAATGTTTGTTCCTAC
	Reverse	GGTGTGTGAGAGCATGGATAG
HPSE ChIP 2	Forward	GACACTTCACATCCCGATTCT
	Reverse	ACTTGTGTTCTCTTCCCTTCTC
HPSE ChIP 3	Forward	GGAAAGCGAGCAAGGAAGTA
	Reverse	ATCCCTCCCACTCCTCTTC
HPSE ChIP 4	Forward	TTCTCATCCCTCATCCCTCTC
	Reverse	TTTAGAGTGGGCGCTAAACAA
HLA-F ChIP 1	Forward	TTTGACTCACAGTAGCAGGAC
	Reverse	CCACTCTGTCACTCCTGAATAG
HLA-F ChIP 2	Forward	GGTGGCTCATGCCTGTAAT
	Reverse	ACGGGTTTCACCGTGTTAG
HLA-F ChIP 3	Forward	GAAACTGGTCTCTGTCCTATTTCA
	Reverse	TCTCCTTCTCCTTCTCCTTCTC

HLA-F ChIP 4	Forward	TCAATCAAGGGACTGGGATTT
	Reverse	CTCAATGTCTCCCTGAGTCTTC
SELL ChIP 1	Forward	TACTGTGCAGAAGCTCTTTAGTT
	Reverse	GGACGTAGGTATGGGCAAAG
SELL ChIP 2	Forward	GGATGAAGCCAACTTGATTGTG
	Reverse	CAGGCCAATATCCCTGATGAA
SELL ChIP 3	Forward	GCGCTGAGCTTCAGAGTTT
	Reverse	GACCCACTGATGTGAGTCAATG
SELL ChIP 4	Forward	GGAAGAAGAACAGCAACAACAA
	Reverse	TCTCTTCCTTCCCTCCTTTCTA

Table S5. Information for primary antibodies used in this study

Antibodies	Vendors	Cat#
CDYL	Proteintech	17763-1-AP
CDYL2	Abcam	ab183854
H3K9me3	Abcam	ab176916
HP1α	Abcam	ab109028
Fibrillarin	Abcam	ab166630
SC35	Abcam	ab11826
SC35	Abcam	ab204916
NPM	Bethyl	A302-404A
PML	Abcam	ab179466
NONO	Abcam	ab133574
Coilin	Abcam	ab87913
Flag	Abcam	ab205606
Flag	Sigma-Aldrich	F1804/F3165
Vinculin	Sigma-Aldrich	V9131
HA	CST	3724S
